# Virulence of Marek’s disease virus in Japan is linked to polymorphisms in the meq oncogene

**DOI:** 10.1099/jgv.0.002232

**Published:** 2026-02-19

**Authors:** Yoshinosuke Motai, Aoi Kurokawa, Jumpei Sato, Shunsuke Yamagami, Shwe Yee Win, Fumiya Horio, Hikaru Saeki, Naoya Maekawa, Tomohiro Okagawa, Benedikt B. Kaufer, Nikolaus Osterrieder, Mark S. Parcells, Satoru Konnai, Kazuhiko Ohashi, Shiro Murata

**Affiliations:** 1 Department of Disease Control, Faculty of Veterinary Medicine, Hokkaido University, Sapporo, Japan; 2 National Institute of Animal Health, National Agriculture and Food Research Organization, Tsukuba, Japan; 3 Institut für Virologie, Freie Universität Berlin, Berlin, Germany; 4 Tierärztliche Hochschule Hannover, Hannover, Germany; 5 Department of Animal and Food Sciences, University of Delaware, Newark, USA; 6 Institute for Vaccine Research and Development (HU-IVReD), Hokkaido University, Sapporo, Japan; 7 Veterinary Research Unit, International Institute for Zoonosis Control, Hokkaido University, Sapporo, Japan; 8 International Affairs Office, Faculty of Veterinary Medicine, Hokkaido University, Sapporo, Japan

**Keywords:** Marek’s disease virus, Meq, Japan, open-mouth breathing, bronchus-associated lymphoid tissue, *γδ* T cells

## Abstract

Marek’s disease virus (MDV) causes lymphomas and neurological disorders in chickens. Although vaccination largely controls outbreaks, highly virulent strains continue to emerge. The major MDV-encoded oncoprotein is Meq, functions as a transcription factor. Amino acid polymorphisms in Meq have been reported to influence virulence. Despite routine vaccination, MD still occurs sporadically in Japan. Japanese isolates harbour characteristic Meq polymorphisms, but their impact on MDV virulence remains unclear. We investigated the transcriptional regulation by Meq from Japanese isolates and evaluated the pathogenicity of recombinant MDV (rMDV) encoding Meq from the Japanese isolate Nr-c1. Nr-c1-Meq exhibited reduced transrepression and transactivation on viral gene promoters. An rMDV encoding Nr-c1-Meq (vNr-c1-Meq) induced lower mortality and tumourigenicity than an rMDV encoding Meq from the parental very virulent RB-1B strain (vRB-1B). vNr-c1-Meq did not cause visceral tumours or neurological disorders but resulted in distinct clinical signs, including open-mouth breathing. In lymphoid tissues from vNr-c1-Meq-infected chickens, a lower proportion of CD4^+^ T cells, the targets of MDV transformation, and lower viral loads were observed than those in vRB-1B-infected chickens. Histopathological examination revealed increased lymphocyte infiltration in bronchus-associated lymphoid tissues (BALT) in the vNr-c1-Meq group. Additionally, flow cytometric analysis showed elevated *γδ* T-cell proportions, which positively correlated with IFN-*γ* expression in vNr-c1-Meq-infected chickens and were linked to BALT hyperplasia. In comparison to the vRB-1B, vNr-c1-Meq infection resulted in attenuated disease progression and altered clinical signs. These findings suggest that Meq polymorphisms not only influence MD virulence but also clinical presentation.

**Impact Statement**

Marek’s disease virus (MDV) causes tumours and paralysis in chickens and poses a major threat to the global poultry industry. Although MD is controlled by vaccination, MDV continuously evolves to evade vaccine-induced immunity and increased virulence. In Japan, the MD outbreak still occurs sporadically in vaccinated flocks. We focused on the MDV-specific oncoprotein, Meq, which plays a crucial role in tumourigenesis. We identified amino acid polymorphisms in Meq that are characteristic of MDV strains in Japan and examined whether these affect MDV pathogenicity. To address this, we infected chickens with recombinant viruses encoding Meq from Japanese isolates and observed milder disease progression and different clinical signs, such as open-mouth breathing and depression. The recombinant virus harbouring the Japanese Meq did not cause solid tumours but induced lung inflammation. By understanding how Meq variations influence MDV pathogenicity, we aim to contribute to improved disease control in the poultry industry. Our findings provide insight into how these variations affect disease outcomes.

## Data Summary

The authors confirm that all supporting data, code and protocols have been provided within the article or through supplementary data files. Whole-genome sequences of recombinant viruses (accession numbers: vRB-1B, LC849255; vNr-c1-Meq and LC859933) and the ORFs of the *meq* genes used in this study (accession numbers: RB-1B-*meq*, EF523390; Nr-c1-*meq*: LC385871; Gf-c1-*meq*: LC208801) have been deposited in the National Center for Biotechnology Information GenBank.

## Data availability

The genome sequence of the newly generated recombinant virus has been deposited in NCBI GenBank under accession no. LC859933 (vNr-c1-Meq).

## Introduction

*Mardivirus gallidalpha2,* which is generally called Marek’s disease virus (MDV), causes lymphoma and neurological disorders in chickens. MDV induces cytolytic infection in B and T cells during the acute phase and subsequently establishes latency primarily in activated CD4^+^ T cells [1]. MDV can transform latently infected CD4^+^ T cells, leading to lymphoma development in infected chickens [2]. Currently, MD occurrence is effectively controlled using attenuated and apathogenic live vaccines [3]. However, sporadic cases of MD are still reported worldwide [4,10], and the increase in virulence of MDV field strains raises concerns about the risk of future outbreaks. MDV virulence is classified into four groups: mild (m), virulent (v), very virulent (vv) and very virulent plus (vv+) [11]. Although MD vaccines prevent tumour formation, they do not stop virus infection or shedding. These ‘leaky vaccines’ contribute to the persistence and transmission of highly virulent MDV in chickens [12].

The MDV oncogene *meq* is encoded in the internal and terminal repeat long (IRL and TRL) regions of the MDV genome. It is essential for tumourigenesis, as *meq*-deletion viruses do not induce lymphomas in chickens [13]. The Meq protein functions as a transcription factor structurally similar to c-Jun (a member of the AP-1 family) and is involved in tumourigenesis by regulating the expression of both host and viral genes [14,15]. Meq contains an N-terminal proline-glutamine-rich region (Pro/Gln), a basic region (BR), a leucine zipper domain (ZIP) and a C-terminal transactivation domain (TD) [16]. It forms homodimers with itself and heterodimers with basic leucine zipper (bZIP) proteins such as c-Jun, JunB and c-Fos. The dimerization with various partners is required for tumourigenesis [17]. The Meq–c-Jun heterodimer modulates the expression of several genes, such as *JTAP-1*, *HB-EGF*, *JAC*, *STAT-3* and *Bcl-2*, which are upregulated in Meq-transformed cells and are believed to contribute to MDV-mediated transformation [15,18]. The transactivation domain contains distinctive proline-rich regions (PRRs), which are thought to influence the extent of transcriptional regulation [19].

The *meq* gene is among the most diverse MDV genes [20], and its evolutionary rate is comparable to that of RNA viruses [21]. Replacing *meq* in the very virulent RB-1B strain with *meq* genes from different pathotypes altered the virulence of the virus [22], highlighting that amino acid polymorphisms in Meq influence MDV virulence. In the USA, low-virulence strains (mMDV and vMDV) retain four consecutive prolines (PPPP) within the PRRs of Meq, whereas highly virulent strains (vvMDV and vv+MDV) are characterized by substitutions at the second proline in the PPPP sequences at amino acid positions 153, 176 and 217 (PPPP to PXPP) [23,24]. Although the role of these PPPP sequences has yet to be sufficiently determined, disruption of these motifs may be associated with high virulence. Similarly, within the BR, differences in the amino acids at positions 71, 77 and 80 are linked with differences in the virulence of the respective strains. In mMDV and vMDV, Meq is characterized by combinations of alanine, glutamate and tyrosine, or serine, alanine/glutamate and aspartate, whereas in vvMDV and vv+MDV, alanine, lysine and aspartate residues occur at these positions. We have previously shown that the combination of serine, alanine and aspartate at positions 71, 77 and 80 in Meq is associated with the diminished virulence of a recombinant virus of the RB-1B strain, which has the amino acid combination of alanine, lysine and aspartate [25]. In addition, it has been found that the combination of alanine, glutamate and tyrosine at these positions in Meq mitigates the virulence of the RB-1B-based recombinant Marek’s disease virus (rMDV) and alters clinical manifestations, including open-mouth breathing accompanied by hyperplasia of bronchus-associated lymphoid tissues (BALT) [26]. Given that the BR has been established to contribute to DNA-binding specificity [16], these mutations may influence the regulation of Meq-mediated transcription. In most U.S. strains, the residue at position 115 within the ZIP domain is valine, although in some vMDVs, alanine occurs at this position [4]. Although Meq dimerizes via this ZIP domain, the residue at position 115 does not appear to influence hydrophobic contacts [17] or the transactivation activity of Meq [27].

In Japan, MD cases are sporadically reported despite vaccination. Although pathotyping of Japanese isolates has not been established, a previous study found that a Japanese isolate exhibited moderate disease progression compared to the vv +MDV strain in the experimental infection [28]. The findings of genomic analyses have revealed that certain genes of Japanese isolates, including *meq*, *gB*, *gL* and *UL6*, are characterized by unique features [29], and that the *meq* genes are genetically related to those in viruses isolated in Europe, Asia and Africa and are grouped into the same major cluster [29,30]. In addition, *meq* gene sequences from MDV strains isolated throughout Asia have been grouped into four major clusters: China, East Asia, South Asia and Middle East Asia clusters. Notably, however, most of the *meq* genes from Japanese strains are not classified into any of these groups [31]. In the BR of Meq from most Japanese isolates, the amino acid combination at positions 71, 77 and 80 consists of alanine, glutamate and tyrosine [27], which is the same as sequences in vMDV strains isolated in the USA, as well as in the highly virulent strains from China, Europe and Africa [4,8, 23, 31,35]. Moreover, similar to the highly virulent strains from China, the ZIP of Meq in most Japanese isolates has an alanine residue at position 115 [4,31]. Within the Meq sequence of Japanese strains, the PRRs contain a PXPP sequence at position 217, at which alanine is found. This motif is common in most vvMDV and vv+MDV strains in the USA, as well as in Chinese strains. Furthermore, PRRs within the sequences of Meq in Japanese strains contain a unique residue at position 176. However, whereas US strains typically encode proline or alanine at this site, Japanese strains such as Nr-c1 and Gf-c1 generally encode a serine (the most common residue among Japanese isolates) or leucine (Table 1) [27]. Similar differences in the residues at positions 176 and 217 have also been reported in virulent Chinese strains [4,31]. At both the 176 and 217 positions, amino acid substitutions disrupt the PPPP motif, resulting in a reduced number of PPPP sequences. Thus, although the Meq from Japanese strains tends to have characteristics of both high and low virulence strains, its effects on MDV virulence have yet to be determined. Accordingly, in this study, we sought to analyse Meq from Japanese strains with respect to its transrepression and transactivation activity and examined its influence on the pathogenicity of MDV.

**Table 1.** Distinct polymorphisms in meq of Japanese strains of Marek’s disease virus

Strain	Accession no.	Isolation	Virulence	Basic region			Leucine zipper	Transactivation domain		
				71	77	80	115	153	176	217
Nr-c1	LC385871	Japan	n.d.	**A**	**E**	**Y**	**A**	**P**	**S**	**A**
Gf-c1	LC208801	Japan	n.d.	**A**	**E**	**Y**	**A**	**P**	**L**	**A**
GX0101	JX844666	China	vv	**A**	**E**	**Y**	**A**	**P**	R	**A**
ATE	AY571784	Hungary	vv	**A**	**E**	**Y**	V	**P**	P	P
LEC-LG	OR592064	Nigeria	n.d.	**A**	**E**	**Y**	V	**P**	A	**A**
N	AY362718	USA	vv+	**A**	K	D	V	Q	A	**A**
RB-1B	EF523390	USA	vv	**A**	K	D	V	**P**	P	P
567	AY362709	USA	v	**A**	**E**	**Y**	V	**P**	P	**A**
CVI988	AY243333-8	Netherlands	m	S	**E**	D	V	**P**	P	P

Adopted from [23,27, 32]. n.d., not determined. Amino acid positions are shown in Meq, consisting of 339 amino acids. Bold letters indicate the same amino acid as Meq of Japanese isolates.

## Methods

### Cells

Chicken embryo fibroblasts (CEFs) were prepared from 10-day-old fertilized eggs (Iwamura Hatchery Co. Ltd., Shibata, Japan) as described previously [36]. CEFs were cultured in Eagle’s Minimal Essential Medium (Nissui Pharmaceutical Co., Ltd., Tokyo, Japan) supplemented with 10 % bovine calf serum (Sigma-Aldrich Fine Chemicals Co., MO, USA), 10 % tryptose phosphate broth (Difco Laboratories, Detroit, MI, USA), 0.03 % l-glutamine, 100 U ml^−1^ penicillin, 100 µg ml^−1^ streptomycin Pen Strep Glutamine (100×) (Thermo Fisher Scientific, MA, USA) and 0.1 % NaHCO₃ at 37 °C under 5 % CO₂.

DF-1 cells, a chicken fibroblast cell line, were cultured in Dulbecco’s Modified Eagle’s Medium (DMEM) (Fujifilm Wako Pure Chemicals Co.) supplemented with 10 % FBS (MP Biomedicals, Inc., CA, USA) at 39 °C under 5 % CO₂.

### Plasmids

Meq-expression plasmids were constructed as previously described [25]. The ORFs of the *meq* genes derived from RB-1B (accession number: EF523390), Nr-c1 (accession number: LC385871) and Gf-c1 (accession number: LC208801) were amplified by PCR using primers with EcoRI or NotI sites at the 5′-end of each primer, as shown in Table S2 (available in the online Supplementary Material), and cloned into the pCI-neo vector (Promega, Madison, WI, USA). Point mutations were introduced into the *meq* genes of RB-1B and Nr-c1, cloned into the pCI-neo vector by site-directed mutagenesis [37]. The primers used for mutagenesis are shown in Table S2. The Meq variants with mutations were designated as RB-1B (P176S), RB-1B (P217A), RB-1B (P176S, P217A), Nr-c1 (S176P), Nr-c1 (A217P) and Nr-c1 (S176P, A217P).

For the reporter assay, the c-Jun expression plasmid was prepared as previously reported [38]. The promoter regions of pp38 and Meq were cloned into the pGL-3 basic vector (Promega), termed pGL3b-pp38 and -Meq, respectively, as reporter plasmids. The control reporter plasmid pRL-TK (Promega), expressing *Renilla* luciferase, was used to standardize transfection efficiency.

### Dual luciferase reporter assay

DF-1 cells were cultured at 2×10⁵ cells well^−1^ at 39 °C for 24 h. To assess transactivation activity on the Meq promoter, cells in each well were transfected with 300 ng of Meq and c-Jun expression plasmids, 500 ng of reporter plasmids and 10 ng of control pRL-TK (Promega) using Lipofectamine 2000 (Thermo Fisher Scientific), according to the manufacturer’s instructions. For the pp38 promoter assay, cells were transfected with 300 ng of Meq expression plasmids, 500 ng of reporter plasmids and 10 ng of pRL-TK in the same manner. Transfected cells were lysed 24 h after transfection using 1×Passive Lysis Buffer (Promega), and luciferase activity was measured using the Dual-Luciferase Reporter Assay System (Promega) and Phelios AB-2350 (ATTO Corp., Tokyo, Japan). The luminescence intensity of firefly luciferase from the reporter plasmid was normalized to *Renilla* luciferase activity from the control plasmid. Results are presented relative to the luciferase activity in cells transfected with the pCI-neo vector.

### Soft agar colony formation assay

DF-1 cells were transfected with each Meq-expression plasmid using Lipofectamine 2000 (Thermo Fisher Scientific). Meq-expressing cells were then selected after culturing for 10 days with 1 % G418, Geneticin (Thermo Fisher Scientific) and 10 % FBS containing DMEM. After recovery of cell number, cells were seeded at 5×10^4^ cells per well in 6-well plates containing 0.35 % Select Agar (Thermo Fisher Scientific) in 10 % FBS-DMEM and a layer of 0.7 % Select Agar in 10 % FBS-DMEM and incubated for 10 days. Non-transfected, non-selected DF-1 and pCI-neo vector-transfected cells were cultured as negative controls. The number of colonies with diameters >0.1 mm was counted in six fields of each well using ImageJ (https://imagej.net/ij/). Total colony numbers in each well were normalized to the average number of non-transfected DF-1 control group. Two independent experiments were conducted in triplicate.

### Western blotting

Briefly, 5.0×10^4^ Meq-expressing DF-1 cells were collected and lysed with 1 : 1 of PBS and 2× Laemmli Sample Buffer (Bio-Rad) containing 5 % 2-mercaptoethanol (Sigma-Aldrich). Samples were denatured at 95 °C for 5 min and electrophoresed in a 10 % SDS-PAGE. The samples were then transferred to an Immobilon-P Transfer Membrane (Merck Millipore, Burlington, MA, USA). The membrane was blocked with 3 % skimmed milk overnight and incubated with the rabbit anti-Meq antisera (20 µg ml^−1^) [39] or Anti-Actin Antibody, clone C4 (20 µg ml^−1^) as an internal control for 1 h at room temperature. The membrane was washed three times with PBS containing 0.5 % Tween 20 (PBS-T) and incubated with anti-rabbit IgG secondary antibody conjugated with HRP (Promega) or anti-mouse IgG_1_ secondary antibody conjugated with HRP (Thermo Fisher Scientific) for 30 min at room temperature. After washing with PBS-T, the membrane was treated with Immobilon Western Chemiluminescent HRP Substrate (Merck Millipore), and the luminescence was imaged using Ex-Capture MG (Atto Corp.)

### Generation of recombinant viruses

The Nr-c1-*meq* was inserted into the RB-1B genome using two-step Red-mediated mutagenesis as previously described [40]. The *meq* gene was replaced in pRB-1B_ΔIRL [41], which harbours a deletion of most of the IRL region [42], which is rapidly restored during virus reconstitution as shown previously. Subsequently, the native *meq* gene was partially deleted (pRB-1B_ΔIRL_Δ*meq*) [43], leaving only the highly conserved N and C terminal ends (40 bp) behind for homologue recombination. The Nr-c1-*meq* and RB-1B-*meq* were inserted into pRB-1B_ΔIRL_Δ*meq*, and the resulting clones were confirmed by restriction fragment length polymorphism (RFLP) analyses, PCR and Sanger sequencing of the *meq* locus. rMDVs were reconstituted by co-transfecting BAC plasmids and the pCAGGS-Cre plasmid (Gene Bridges GmbH, Heidelberg, Germany) into CEFs. IRL restoration and BAC sequence excision were verified by PCR [41]. In addition, the absence of unintended mutations elsewhere in the viral genome, excluding *meq*, was confirmed by whole-genome sequencing (accession numbers: vRB-1B, LC849255; vNr-c1-Meq, LC859933) as described previously [44].

### Growth kinetics of rMDVs *in vitro*

Monolayer CEFs were infected with 50 p.f.u. well^−1^ of each rMDV, and infected CEFs from four wells per group were collected daily for 5 days. The viral loads in total cellular DNA were analysed by quantitative PCR (qPCR).

### Analysis of viral pathogenicity *in vivo*

Fertilized eggs from conventional White Leghorn chickens (Iwamura Hatchery Co. Ltd.) were hatched in the laboratory and raised in isolators, specifically the Direct Micro Bio-clean Capsule Unit (Tokiwa Kagaku Kikai Co. Ltd., Tokyo, Japan), within the ABSL2 biosafety room at the animal facility of the Faculty of Veterinary Medicine. One-day-old chicks were randomly divided into three groups and housed separately. The day-old chicks were intraperitoneally inoculated with 5,000 p.f.u. of each rMDV or PBS as a negative control. Chickens in each experimental group were housed in separate isolators, with the number of birds per unit adjusted according to their body-weight growth. After viral inoculation, the animals were monitored daily for clinical signs of MD until the end of the experiment. Additionally, veterinarians at the animal facility assessed the health condition of chickens daily throughout the experimental period. All animal experiments were performed by trained personnel in accordance with the regulations of Hokkaido University. All chickens were euthanized at a humane endpoint, defined as the onset of neurological signs or clinical indicators such as depression, on the day of the diagnosis. All chickens were euthanized via heparinized whole blood collection from the heart under deep general anaesthesia induced by isoflurane inhalation (Zoetis Japan, Tokyo, Japan). All data that could be linked to individual animals in the animal experiments 1 and 2 are presented in Tables S3 and S4, respectively.

### Animal experiment 1

Chickens were randomly divided into control (*n*=20), vRB-1B-infected (*n*=34) and vNr-c1-Meq-infected groups (*n*=33). Four chickens per group were randomly selected and euthanized, and their spleen, thymus and whole blood were collected at 7, 14, 28 and 35 days post-infection (dpi) to monitor viral loads. Additionally, four chickens per group were randomly selected each time from the remaining birds, and whole blood was collected at 42, 49 and 56 dpi.

Clinical signs of remaining chickens in the control (*n*=4), vRB-1B-infected (*n*=18) and vNr-c1-Meq-infected groups (*n*=17) were monitored daily for pathogenicity analysis. Pathogenicity of rMDVs was evaluated by monitoring clinical signs of MD and tumour incidence. Chickens showing clinical signs were euthanized at humane endpoints, and Kaplan–Meier survival curves were generated to compare disease incidence (survival rate) in infected chickens. The remaining chickens were euthanized at 63 dpi. Gross tumour lesions were examined in all chickens, and whole blood, spleen and thymus samples were collected to analyse viral loads and T-cell subset proportions.

### Animal experiment 2

Clinical signs of chickens in the control group (*n*=5), vRB-1B (*n*=20) and vNr-c1-Meq (*n*=22) groups were monitored daily for further pathogenicity analysis. Five chickens per group were randomly selected each time, and whole blood was collected at 7, 14, 28, 35, 42 and 49 dpi to periodically monitor viral loads. Whole blood was also used for re-isolation of the virus at 7, 14, 28 and 49 dpi. Chickens that showed clinical signs of MD during the experiment were euthanized, and the remaining chickens were euthanized at 56 dpi. Gross tumour lesions were examined in all chickens. Lungs were collected to analyse viral loads and lymphocyte proportion, and plasma was collected to measure IFN-*γ* concentration. Additionally, spleen, bursa of Fabricius, thymus, lung, proventriculus, brain, cervical tissue and tumour were collected from two control chickens, three vRB-1B-infected chickens and five vNr-c1-Meq-infected chickens for histopathological analysis.

### DNA extraction

Total cellular DNA was extracted from rMDV-infected CEFs, whole blood and tissues using the DNeasy Blood and Tissue Kit (QIAGEN, Hilden, Germany) according to the manufacturer’s instructions. The resultant DNA samples were treated with RNase A (Merck Millipore).

### Quantitative PCR

qPCR and real-time qPCR were performed using TB Green Premix DimerEraser (Takara Bio Inc.) and LightCycler 96 system (Roche Diagnostics). qPCR was conducted using primers for *infected cell protein 4* (*ICP4*) as a target gene and *chicken inducible nitric oxide synthase* as a reference gene to quantify the viral loads. Real-time qPCR was performed using primers to analyse mRNA expression of IFN-*γ*. For the real-time qPCR analysis, the mRNA expression of *chicken β-actin* was also analysed as a reference gene. PCR was performed for 40 cycles after denaturation at 95 °C for 30 s, each cycle consisting of denaturation at 95 °C for 5 s, annealing at 55 °C for 30 s and extension at 72 °C for 30 s. After the PCR reaction was completed, the melting temperature of the PCR products was confirmed by increasing the temperature from 65 to 95 °C at 0.11 °C/s for melting curve analysis to confirm product specificity. Viral loads and mRNA expression are indicated as ratios between each target and the reference gene. The primers used for qPCR and real-time qPCR analyses are shown in Table S2. Three independent experiments were performed.

### Flow cytometric analysis

Spleens, thymuses and lungs were dissected and strained using EASYstrainer 40 µm (Greiner Bio-One GmbH, Kremsmünster, Austria) to obtain cell suspensions, and mononuclear cells were isolated by density gradient centrifugation using Percoll solution (GE Healthcare). To prevent nonspecific antibody reactions, 5×10⁶ cells were incubated with PBS containing 10 % chicken serum at 25 °C for 30 min after washing with RPMI-1640 medium (Sigma-Aldrich, St. Louis, MO, USA). The cells were reacted with 2 µg ml^−1^ of Mouse Anti-Chicken CD4-PE/CY7 antibody (CT-4) (Southern Biotech, Birmingham, AL, USA), 2 µg ml^−1^ of Mouse Anti-Chicken CD8b-FITC (EP42) (Southern Biotech), 2 µg ml^−1^ of Mouse Anti-Chicken TCRγδ-PE (TCR-1) (Southern Biotech) and 2 µg ml^−1^ of Mouse Anti-Chicken CD3-UNLB (CT-3) (Southern Biotech) conjugated with PerCP/Cy5.5 (Abcam, Cambridge, UK) at 4 °C for 30 min. For lung samples, cells were also reacted with 2 µg ml^−1^ of Mouse Anti-Chicken Bu-1-PE (Southern Biotech). For Meq expression analysis, splenocytes were also reacted with anti-Meq mAb [45] conjugated with APC (Abcam, UK). Dead cells were stained using Fixable Viability Dye eFluor780 (Thermo Fisher Scientific). The stained cells were washed twice with PBS containing 1 % bovine serum albumin and analysed using a FACS Lyric flow cytometer (BD Biosciences, Franklin Lakes, NJ, USA). Gating strategies were constructed for lymphocyte subset analysis (Fig. S7a–c).

### Virus reactivation assay

To assess the titres of infective viruses, rMDVs were rescued from PBMCs, which were collected from five chickens per group at 7, 14, 28 and 49 dpi in animal experiment 2, and isolated by density gradient centrifugation using Percoll solution (GE Healthcare, Chicago, IL, USA). We inoculated 10⁶ PBMCs onto monolayered CEFs and cultured them for 7 days. The samples from each individual at each time point were subjected to three wells. Plaque numbers were counted, and the results were expressed as ratios to 10⁶ PBMCs or ratios to 1 ml of whole blood.

### Histopathological analysis

Histopathological analysis was performed according to a standard protocol for HE staining and the immunohistochemistry (IHC) protocol previously reported [46]. mAbs against Meq [45] and pp38, which were recently generated in the same manner as the anti-Meq mAbs, were used for IHC analysis. Briefly, recombinant pp38 was prepared from *Spodoptera frugiperda* 21 cells infected with recombinant baculovirus harbouring the *pp38* gene of the Md5 strain. Two 7-week-old BALB/c mice (Japan SLC, Shizuoka, Japan) were intraperitoneally immunized with recombinant pp38 antigen (10 µg per dose) mixed with an aluminium hydroxide-based adjuvant (2 mg in 100 µl per dose) three times at 2-week intervals. The spleen cells of the immunized mice were fused with mouse myeloma P3U1 cells, and the resultant hybridomas secreting anti-recombinant pp38 IgG mAbs were screened via indirect ELISA and cloned twice using single-cell sorting.

Organs collected in animal experiment 2 were fixed in 10 % neutral-buffered formalin (Fujifilm Wako Pure Chemical Co.) for 2 days and embedded in paraffin. The sections were deparaffinized and incubated in 0.3 % H₂O₂ in methanol for 20 min at room temperature (20–26 °C) to block endogenous peroxidase activity. After high-temperature-induced antigen retrieval at pH 9.0, the sections were cooled at room temperature for 30 min. To reduce nonspecific antibody reactions, the sections were treated with a 5 % skim milk solution (Fujifilm Wako Pure Chemical Co.) for 20 min. The sections were incubated with supernatants from hybridoma cell cultures containing anti-Meq mAbs (1.4 mg ml^−1^) [47] or anti-pp38 mAbs (4 µg ml^−1^) as primary antibodies at 4 °C for 20–24 h. After rinsing with PBS, the sections were treated with a horseradish peroxidase polymer-based secondary antibody reagent (Histofine Simple Stain MAX PO (M) kit, Nichirei Bioscience Inc., Tokyo, Japan) for 45 min at room temperature (20–26 °C). Antigen-antibody reactions were visualized using the 3,3′-diaminobenzidine tetrahydrochloride substrate kit (Nichirei Bioscience Inc.). The slides were counterstained with Mayer’s hematoxylin (Muto Pure Chemicals, Tokyo, Japan).

### mRNA extraction

Mononuclear cells that were isolated by density gradient centrifugation using Percoll solution (GE Healthcare) from the spleens and lungs of chickens in animal experiment 2 were suspended in TRI Reagent (Molecular Research Centre, Inc., Cincinnati, OH, USA), and total cellular RNA was extracted according to the manufacturer’s instructions. Residual DNA was removed by treatment with DNase I (Promega). cDNA was synthesized in a mixture containing PrimeScript Reverse Transcriptase (Takara Bio Inc.) and Oligo dT Primer (Takara Bio Inc.).

### Quantification of IFN-*γ* concentration in plasma by ELISA

IFN-*γ* concentrations in plasma were quantified using the Chicken IFN-*γ* CytoSet (Thermo Fisher Scientific) following the manufacturer’s instructions. Capture antibodies were coated onto Nunc Immuno Plate F96 Maxisorp (Thermo Fisher Scientific). Absorbance at 450 nm was measured using an MTP-900Lab multiple reader (CORONA ELECTRIC, Ibaraki, Japan).

### PCR screening for respiratory pathogens

DNA extracted from the lungs of vNr-c1-Meq-infected chickens was used to screen for *Mycoplasma gallisepticum*, *Mycoplasma synoviae*, *Pasteurella multocida*, *Bordetella avium*, *Escherichia coli* and infectious laryngotracheitis virus by PCR. Subsequently, cDNA synthesized from lung RNA samples was used to test for infectious bronchitis virus, Newcastle disease virus and avian influenza virus. All PCR assays were performed using TaKaRa Ex Taq (Takara Bio Inc.) in SimpliAmp Thermal Cycler (Thermo Fisher Scientific). All primer sequences used are shown in Table S2. The cycling conditions were as follows: initial denaturation at 98 °C for 2 min, followed by 35 cycles of denaturation at 98 °C for 10 s, annealing at 54 °C for 30 s and extension at 72 °C for 2 min. The amplified PCR products were analysed by 1.5 % agarose gel electrophoresis, and the specific bands were used for pathogen detection.

### Statistical analysis

Statistical analyses were performed using R version 4.4.0 (https://www.r-project.org/). The levels of transrepression and transactivation activities and colony numbers in the soft agar colony formation assay between groups were analysed by the Tukey–Kramer test. The Kruskal–Wallis test was performed to compare the growth kinetics of rMDVs *in vitro*. The Steel–Dwass test was performed to compare viral loads, weights of lymphoid organs, proportions of T cell subsets and mRNA expression in multiple groups. The Mann–Whitney U test was conducted to compare the growth kinetics of rMDVs *in vivo*, plaque numbers in the reactivation assay and viral loads in animal experiment 2. The log-rank test was conducted to compare disease incidence (survival rate). Disease incidence and tumour incidence were compared by Fisher’s exact test. *P*<0.05 was considered statistically significant.

## Results

### Effects of polymorphisms in Meq of Japanese isolates on the transcriptional activities

**Fig. 1.**
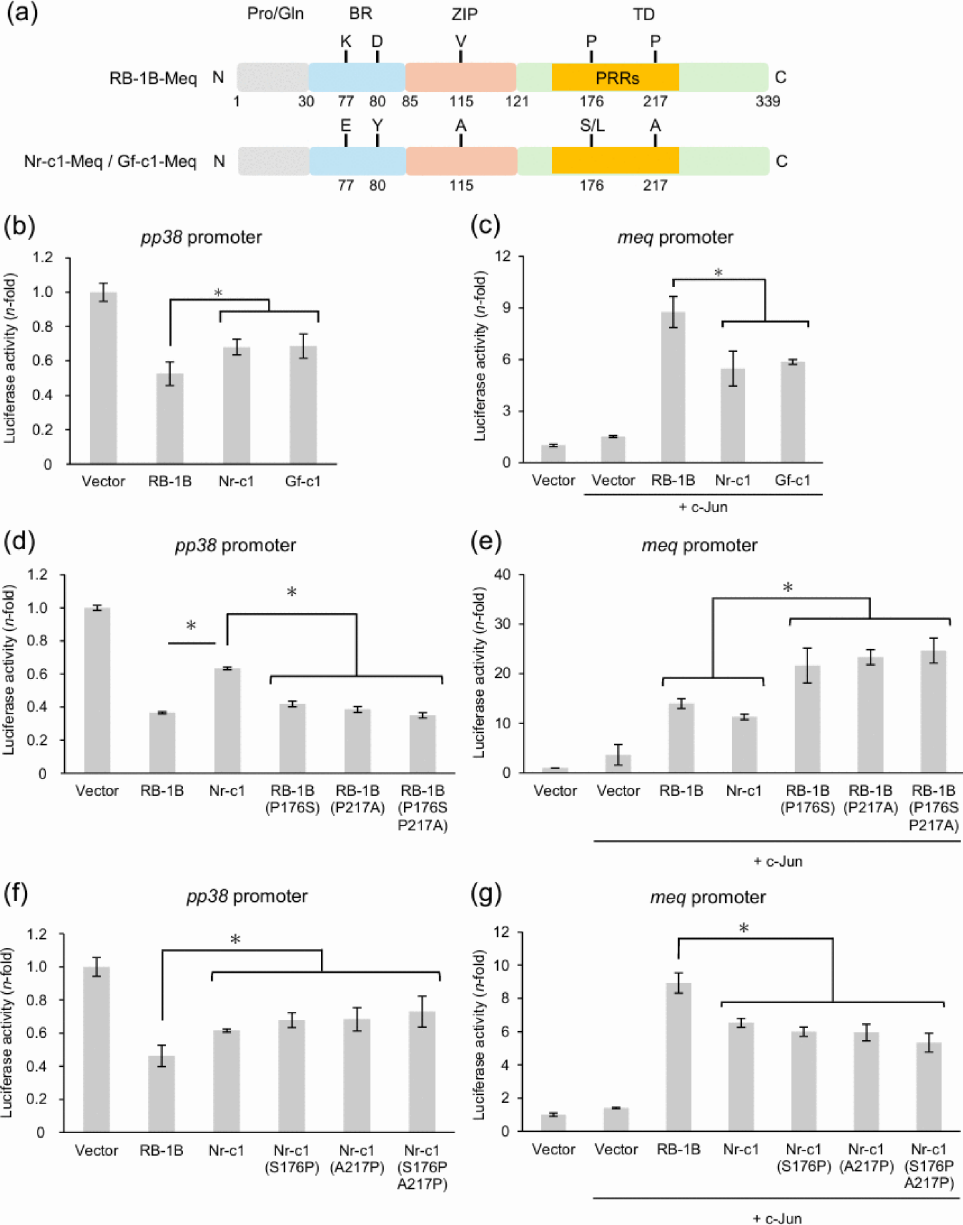
Effects of polymorphisms in Meq of Japanese isolates on the transrepression of the *pp38* promoter and the transactivation activities on the *meq* promoter. (**a**) Domain map of Meq proteins of RB-1B and Nr-c1/Gf-c1 strains. The Meq protein consists of the N-terminal proline-glutamine-rich region (Pro/Gln), the basic region (BR), the ZIP and the C-terminal TD, which includes proline-rich repeats (PRRs). Amino acid differences between RB-1B-Meq and Nr-c1-Meq are located at positions 77, 80, 115, 176 and 217. (**b**) and (**c**) Transcriptional regulation by Meq from Japanese isolates, Nr-c1 and Gf-c1, was compared with RB-1B-Meq using reporter assays targeting the promoters of (**b**) *pp38* and (**c**) *meq*. (**d**) Transrepression on the *pp38* promoter and (**e**) transactivation activity on the *meq* promoter by RB-1B-Meq with polymorphisms in the transactivation domain of Nr-c1-Meq relative to RB-1B (proline-to-serine substitution at position 176, P176S; proline-to-alanine substitution at position 217, P217A; or both, P176S P217A) were analysed. (**f**) Transrepression on the *pp38* promoter and (**g**) transactivation activity on the *meq* promoter by Nr-c1-Meq matched to the same amino acids as RB-1B-Meq (serine-to-proline substitution at position 176, S176P; alanine-to-proline substitution at position 217, A217P; or both, S176P A217P) were analysed. The transactivation activity at the *meq* promoter was assessed in the presence of the c-Jun expression plasmid. Firefly luciferase luminescence was normalized to *Renilla* luciferase activity and expressed relative to the luciferase values in cells transfected with the empty pCI-neo vector. Three independent experiments were conducted in triplicate. Data are shown from one representative experiment. Error bars represent standard deviations. Asterisks indicate significant differences (*P<0.05; Tukey–Kramer test).

In this study, we compared Meq characteristics between Japanese isolates and RB-1B to determine how Meq from Japan affects the transcriptional regulation and MDV virulence. The amino acid sequences of Meq from Japanese isolates differ from RB-1B-Meq at five positions (Fig. 1a). Nr-c1-Meq contains glutamate and tyrosine at positions 77 and 80 in the BR, whereas RB-1B-Meq has lysine/serine and aspartic acid at these sites. In the PPPP sequences of the PRRs, Meq from Japanese isolates encodes serine and alanine at positions 176 and 217, while RB-1B-Meq encodes proline at both sites. Additionally, a difference at position 115 in the ZIP domain was observed (alanine in Meq from Japanese isolates and valine in RB-1B-Meq).

To analyse the effects of polymorphisms in Meq from Japanese isolates on transcriptional regulation, we conducted reporter assays targeting two promoters, *pp38* and *meq*. The Meq/Meq homodimer suppresses *pp38* promoter activity [14], while the Meq/c-Jun heterodimer enhances transactivation activity at the *meq* promoter [19]. Transrepression of the *pp38* promoter by Meq from Japanese strains was weaker than that of RB-1B-Meq (Fig. 1b). Additionally, transactivation of the *meq* promoter by Meq from Japanese strains was lower than that of RB-1B-Meq (Fig. 1c). No difference in promoter response was observed between the two Japanese Meq proteins (Fig. 1b and c). Thus, Meq from Japanese isolates showed weaker transrepression and transactivation activities than RB-1B-Meq.

Subsequently, we assessed the effects of individual amino acid differences in the PRRs between the two strains on transcriptional regulation. Amino acid substitutions at positions 176 and 217 were introduced into Nr-c1-Meq and RB-1B-Meq, respectively. Compared to wild-type RB-1B-Meq, substitutions at these sites (proline-to-serine at 176, P176S; proline-to-alanine at 217, P217A; or both, P176S, P217A) showed no difference in *pp38* promoter transrepression (Fig. 1d). All RB-1B-Meq constructs showed stronger transrepression than Nr-c1-Meq constructs (Fig. 1d). Compared to wild-type RB-1B-Meq and Nr-c1-Meq, all RB-1B-Meq constructs with substitutions exhibited enhanced transactivation activity at the *meq* promoter (Fig. 1e). In contrast, substitutions in Nr-c1-Meq (S176P, A217P and both) reduced *pp38* promoter transrepression compared to wild-type RB-1B-Meq, whereas no difference was observed compared to wild-type Nr-c1-Meq (Fig. 1f). All Nr-c1-Meq constructs exhibited lower transactivation activity at the *meq* promoter than the wild-type RB-1B-Meq (Fig. 1g). Collectively, these findings suggest that transcriptional regulation of Meq strongly depends on the bZIP sequence and that polymorphisms in PRRs may also influence transactivation activity in a manner dependent on the bZIP sequence.

### Transformation capacity of the Meq from Japanese isolates

**Fig. 2.**
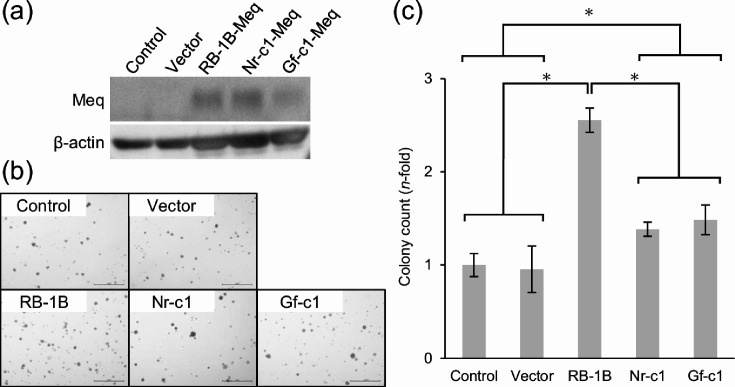
Transformation capacity of Meq from Japanese isolates. (**a**) Meq expression in the stable cell lines of RB-1B-Meq, Nr-c1-Meq and Gf-c1-Meq cells. Non-transfected DF-1 (Control) and empty vector-transfected cells (Vector) were loaded as negative controls. (**b**) Meq-expressing cells were cultured in soft agar medium for 10 days. Representative fields of each group are shown. (**c**) Relative colony numbers in each Meq-expressing cell were counted in six fields from each well. Total colony numbers were normalized to the mean of the control group. Two independent experiments were conducted in triplicate. Data shown are from one representative experiment. Error bars represent standard deviations. Asterisks indicate significant differences (*P<0.05; Tukey–Kramer test).

We performed a soft agar colony formation assay using stable cell lines expressing RB-1B-Meq, Nr-c1-Meq or Gf-c1-Meq to assess the transformation capacity of Meq in the Japanese isolates. Non-transfected DF-1 cells and empty vector-transfected cells were used as controls. After G418 selection, comparable levels of Meq protein were detected among the Meq-expressing cell lines (Figs 2a and S1a, b). Cells were then cultured in soft agar medium for 10 days, and colony numbers were quantified. Nr-c1-Meq- and Gf-c1-Meq-expressing cells exhibited similar colony numbers, which were significantly higher than negative control cells and empty vector-transfected cells (Fig. 2b and c). However, the colony number of cells expressing Meq from Japanese isolates was lower than that of the RB-1B-Meq-expressing cells (Fig. 2b and c). These results indicate that Meq from Japanese isolates has a weaker transformation capacity compared to RB-1B-Meq.

### Generation and characterization of rMDV encoding Nr-c1-Meq

**Fig. 3.**
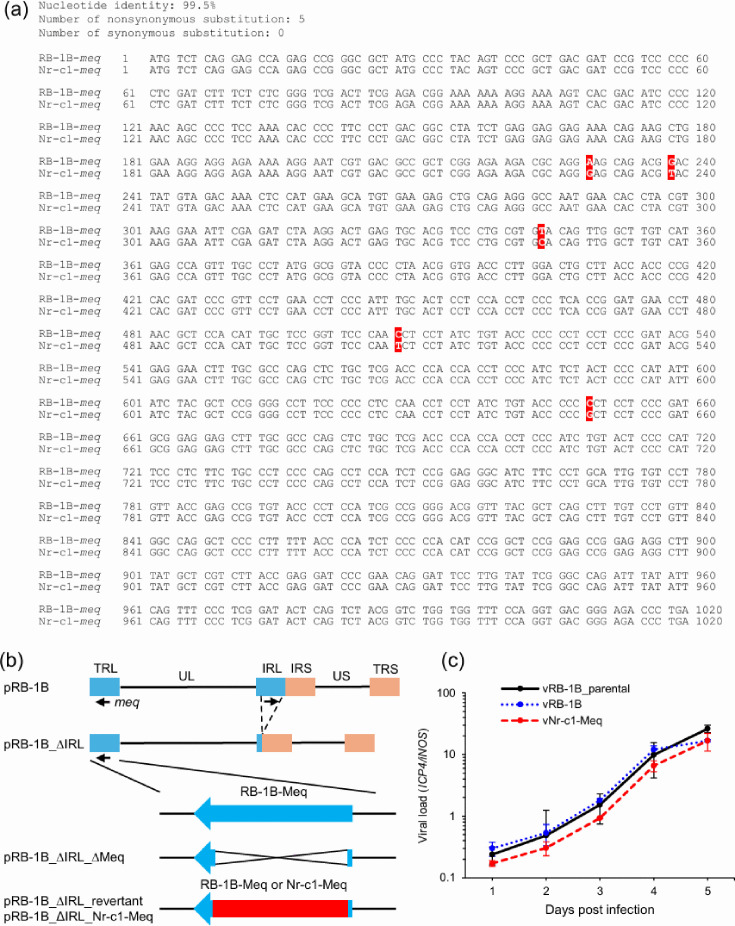
Generation of recombinant MDV encoding Meq of Japanese isolates. (**a**) Alignment of nucleotide sequences between RB-1B-*meq* and Nr-c1-*meq*. Differences in nucleotide sequences are highlighted in red. (**b**) Schematic diagram of the genetic recombination of MDV. pRB-1B, the whole genome of RB-1B cloned as a bacterial artificial chromosome (BAC); pRB-1B_ΔIRL, a BAC plasmid with most of the IRL deleted; pRB-1B_ΔIRL_ ΔMeq, a BAC plasmid with most of the meq gene in TRL deleted in pRB-1B_ΔIRL; pRB-1B_ΔIRL_ revertant and pRB-1B_ΔIRL_Nr-c1-Meq, BAC plasmids in which *meq* from RB-1B or Nr-c1 was inserted into the meq locus in TRL in pRB-1B_ΔIRL_ΔMeq. (**c**) Growth kinetics of each rMDV in infected CEFs were analysed by qPCR. Error bars indicate standard deviations. Statistical analysis was performed using the Kruskal–Wallis test.

An RB-1B-based rMDV encoding Nr-c1-Meq was generated to assess how the Meq amino acid sequence in Japanese strains affects MDV virulence. The nucleotide sequence of Nr-c1-*meq* showed a 99.5 % similarity with the sequence of RB-1B-*meq*, with five nucleotide differences, all of which were non-synonymous substitutions (Fig. 3a). The Nr-c1-*meq* gene was inserted into the RB-1B genome using the bacterial artificial chromosome (BAC)-based genetic system [40,48]. Most of the IRL region in the RB-1B genome cloned as a BAC plasmid (pRB-1B) [42] was deleted (pRB-1B_ΔIRL) [41], and the native *meq* gene in the TRL was partially deleted (pRB-1B_ΔIRL_Δ*meq*) [43]. The Nr-c1-*meq* or RB-1B-*meq* gene was inserted into the *meq* locus in the TRL of pRB-1B_ΔIRL_Δ*meq* (Fig. 3b). Correct insertion of the *meq* gene was confirmed by RFLP analysis and Sanger sequencing (data not shown). rMDVs encoding Nr-c1-Meq or RB-1B-Meq were reconstituted by transfecting each BAC plasmid into CEFs. IRL restoration and BAC sequence excision were confirmed by PCR (data not shown) [41]. The absence of unrelated mutations was verified by whole-genome sequencing (accession number: LC859933). The *in vitro* replication kinetics of the recombinant viruses (vNr-c1-Meq and vRB-1B) were compared to those of the parental rMDV (vRB-1B_parental). No significant differences in viral loads among rMDVs were observed (Fig. 3c), indicating that Nr-c1-Meq does not alter viral replication *in vitro*.

### Pathogenicity of vNr-c1-Meq

**Fig. 4.**
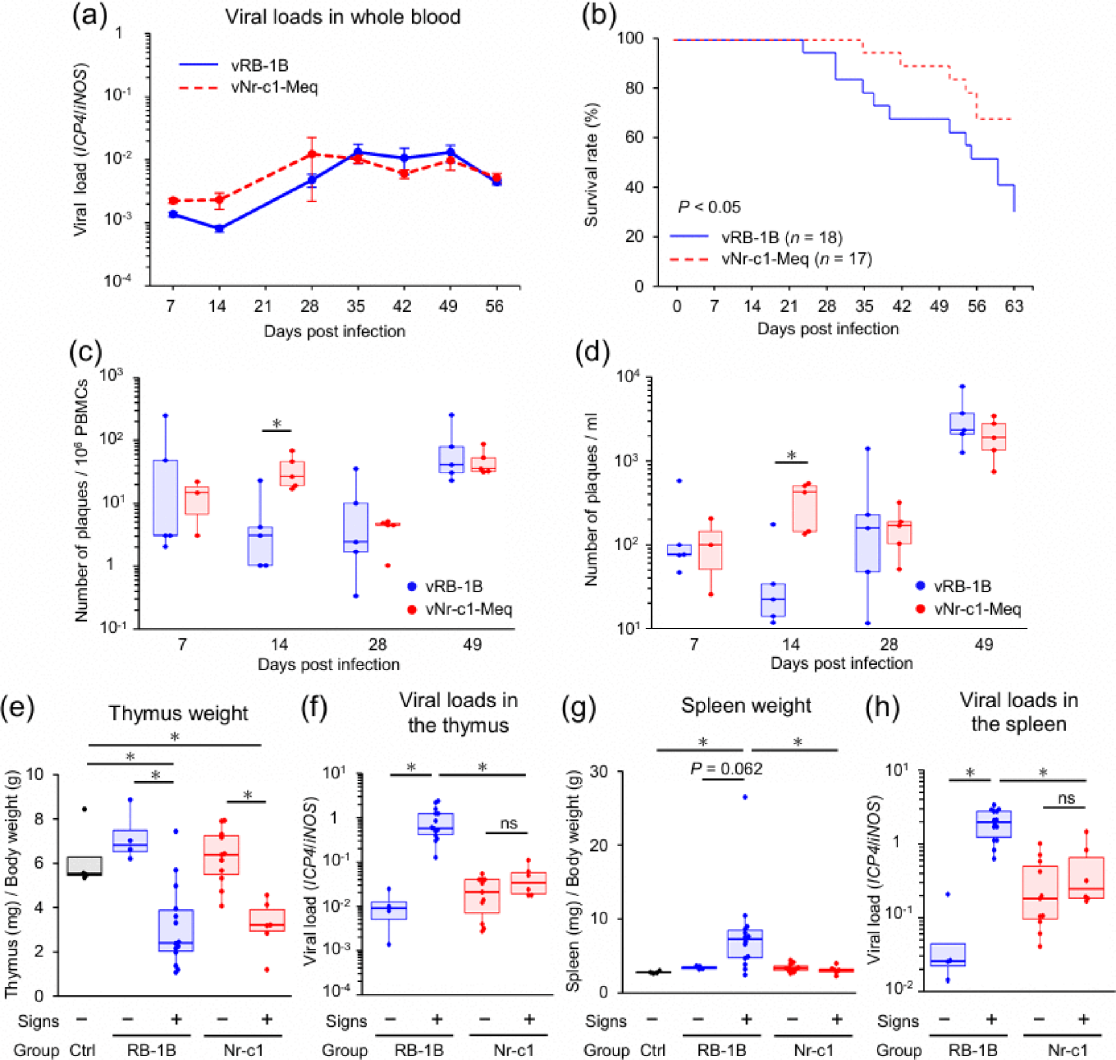
Pathogenicity of vNr-c1-Meq in animal experiment 1. (**a**) Growth kinetics of rMDVs in whole blood were analysed by qPCR. Whole blood samples were collected from randomly selected four chickens per group in the animal experiment 1 at each time point. (**b**) Chickens showing clinical signs were euthanized at humane endpoints, and Kaplan–Meier survival curves were generated. The log-rank test was used to compare survival rates between chickens infected with each rMDV in the animal experiment 1. *P*<0.05 was considered statistically significant. (**c**) and (**d**) The number of plaques formed by PBMCs in (**c**) 10^6^ cells and in (**d**) 1 ml of whole blood was counted as the amount of the proliferative rMDVs by reactivation assay. Whole blood samples were obtained from five randomly selected chickens per group in the second animal experiment at each time point. (**e**) and (**g**) The weight of (**e**)the thymus and (**g**)the spleen in rMDV-infected chickens was analysed. Results are presented as ratios of lymphoid organ weight (in mg) to body weight (in g), which were compared between rMDV-infected chickens with clinical signs, such as neurological signs and/or macroscopic tumours (signs+) and those without (signs−). (**f**) and (**h**) Viral loads in (**f**) the thymus and (**h**) the spleen and from rMDV-infected chickens in animal experiment 1 were analysed by qPCR. Viral loads were compared between rMDV-infected chickens with and without disease. (**e**)–(**h**) Samples were collected from chickens in control (*n*=4), vRB-1B signs− (*n*=4), vRB-1B signs+ (*n*=14), vNr-c1-Meq signs- (*n*=11) and vNr-c1-Meq signs+ (*n*=11) in the animal experiment 1. (**c**)–(**h**) Box and whisker plots showing the median (line within the box), interquartile range (box) and minimum and maximum values (whiskers). The dots represent individual data points or outliers. Asterisks indicate significant differences [*P<0.05; (**a**), (**c**), (**d**) Mann–Whitney U test, (**e**)–(**h**) Steel–Dwass test].

Next, we investigated the effects of vNr-c1-Meq on MDV pathogenicity. One-day-old chicks were infected with vNr-c1-Meq or vRB-1B, and viral loads and MD incidence were compared. A humane endpoint was defined as the onset of neurological signs or clinical indicators such as depression with feeding difficulties. No significant differences in viral loads were observed in whole blood, spleen or thymus between groups (Fig. 4a and S2a–c), suggesting that polymorphisms in Nr-c1-Meq do not affect viral replication *in vivo*. However, Kaplan–Meier survival curves showed that chickens infected with vNr-c1-Meq had lower disease incidence than those infected with vRB-1B (Fig. 4b). In vRB-1B-infected chickens, clinical signs like leg paralysis and torticollis were observed in 13 of 18 chickens, with gross tumours in 13 of 18 (Table 2). In contrast, chickens infected with vNr-c1-Meq showed signs such as depression, reduced appetite and open-mouth breathing in 6 of 17 chickens, but no gross visceral tumours developed (Table 2). To validate these observations, we conducted a second independent animal experiment. Although the disease and tumour incidence were lower than those in the first experiment, presumably due to multiple unidentified factors, including the shorter experimental period and environmental factors, we observed similar differences in clinical signs (Figs S3a, Table S1) and no significant difference in viral loads in whole blood (Fig. S3b). However, when viruses were rescued from peripheral blood mononuclear cells (PBMCs), vNr-c1-Meq-infected chickens exhibited higher numbers of plaques than vRB-1B-infected chickens at 14 dpi, in the plaque counts per 10⁶ PBMCs and per 1 ml of blood (Fig. 4c and d). Thus, the vNr-c1-Meq group was characterized by higher viral titres at 14 dpi compared with the vRB-1B group, although replacing the native RB-1B-*meq* with Nr-c1-*meq* altered disease incidence and clinical presentation, which is plausibly attributable to a delay in the establishment of latency in vNr-c1-Meq.

**Table 2.** Comparison of the pathogenicity of rMDVs in the animal experiment 1

Group	Clinical sign* (%)	Tumour incidence (%)
vRB-1B	13/18 (72.2)	13/18 (72.2)
vNr-c1-Meq	6/17 (35.3)	0/17 (0)
*P* value (Fisher’s exact test)	0.044	<0.001

*Number of chickens showed leg paralysis, torticollis or depression.

### Changes in the lymphoid organs of chickens with Marek’s disease

MDV causes thymic atrophy and splenomegaly [49,50]. We compared lymphoid organ weights among infected chickens that developed clinical signs and/or gross tumours during the experimental period (chickens with signs, signs+) and chickens without clinical signs or tumour development by 63 dpi (chickens without signs, signs−) in both infected groups and the uninfected control group. Thymus weight decreased in chickens with signs in both infected groups compared to controls and infected chickens without signs (Fig. 4e). These findings suggest thymus atrophy occurred in MD-positive chickens in both groups. However, unlike thymus weight, viral loads in the thymus did not increase in vNr-c1-Meq-infected chickens with signs, although they did increase in vRB-1B-infected chickens with signs (Fig. 4f). In the vRB-1B-infected group, spleen weight in signs-positive chickens tended to exceed that in signs-negative chickens and was heavier than that of controls and vNr-c1-Meq-infected chickens (Fig. 4g). Conversely, no significant spleen weight increase was observed in vNr-c1-Meq-infected chickens with signs (Fig. 4g). Viral loads in the spleen of vRB-1B-infected chickens with signs were significantly higher than in all other groups, while vNr-c1-Meq-infected chickens showed no difference in spleen viral load between signs-positive and signs-negative individuals (Fig. 4h). Whole blood viral load patterns mirrored these trends (Fig. S2c) (Fig. S2e). These results suggest that tumour cells do not develop or proliferate aggressively in vNr-c1-Meq-infected chickens.

### Changes in CD4^+^ T cells and Meq expression in the lymphoid organs during disease progression

**Fig. 5.**
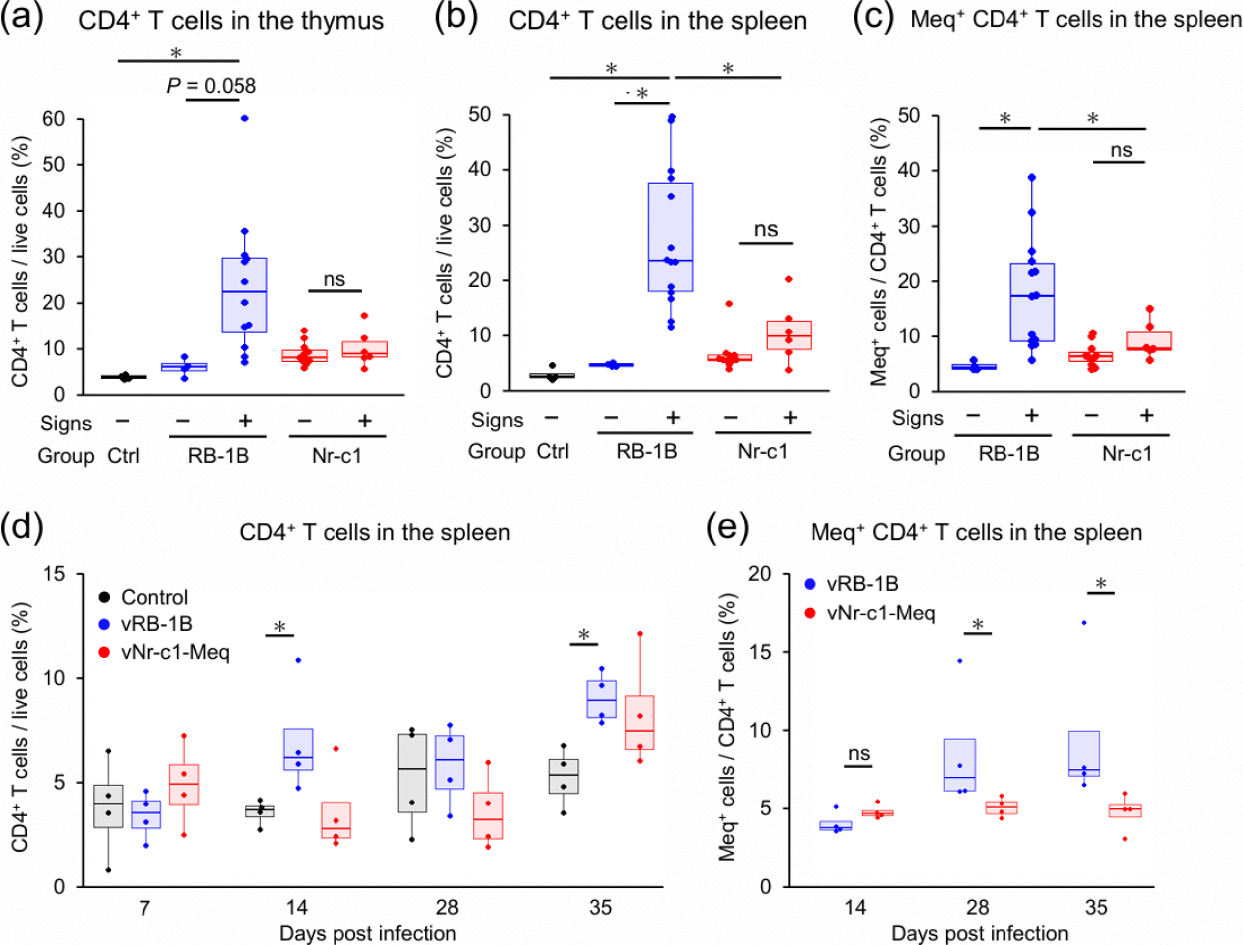
Comparison of the proportion of CD4^+^ T cells and Meq^+^ CD4^+^ T cells in the thymus and spleen. (**a**)–(**c**) The proportion of (**a**) CD4^+^ T cells in the thymus, (**b**) CD4^+^ T cells in the spleen and (**c**) Meq^+^ CD4^+^ T cells in the spleen was analysed at the endpoint. T-cell subset proportions were compared in the spleen and thymus of rMDV-infected chickens with and without clinical signs (signs+and signs−, respectively). Samples from chickens in the control (*n*=4), vRB-1B (signs-, *n*=4), vRB-1B (signs+, *n*=16), vNr-c1-Meq (signs-, *n*=11) and vNr-c1-Meq groups (signs+, *n*=6) were used for these analyses. (**d**) and (**e**) The proportion of (**d**) CD4^+^ T cells and (**e**) Meq^+^ CD4^+^ T cells in the spleen was analysed at 7, 14, 28 and 35 dpi. Four chickens per group were randomly selected at each time point. (**a**)–(**e**) All samples were collected from chickens in animal experiment 1. Box and whisker plots showing the median (line within the box), interquartile range (box) and minimum and maximum values (whiskers). The dots represent individual data points or outliers. Asterisks indicate significant differences [*P<0.05; (**a**)–(**d**) Steel–Dwass test, (**e**) Mann–Whitney U test].

To investigate the progression of disease in both infected groups, we analysed the proportions of CD4^+^ T cells among total live cells in the thymus and spleen, as MD primarily induces the transformation of CD4^+^ T cells [2]. Within the thymus, the proportion of CD4^+^ T cells in the vRB-1B-infected chickens with signs was found to be significantly higher than that in the controls and also tended to be higher than that in the vRB-1B-infected chickens without signs (Fig. 5a). Conversely, we detected no significant increases in CD4^+^ T cells in the vNr-c1-Meq-infected groups, implying limited generation of tumour cells in these chickens (Fig. 5a). Similarly, within the spleen, the proportion of CD4^+^ T cells in vRB-1B-infected chickens with signs was found to be significantly higher than that in the other assessed groups, whereas we observed no significant increases among the vNr-c1-Meq-infected groups (Fig. 5b). Given that the MDV-mediated transformation of CD4^+^ T cells is accompanied by a high expression of Meq, we subsequently analysed the proportion of Meq-expressing cells among CD4^+^ T cells in the spleen [2] and accordingly found that compared with other groups, vRB-1B-infected chickens with signs were characterized by a significantly higher proportions of Meq^+^ cells among the CD4^+^ T cells than those in other groups. In contrast, regardless of clinical signs, we detected no significant increase in the proportion of Meq^+^ cells in the vNr-c1-Meq-infected chickens (Fig. 5c). In addition, at 14 and 35 dpi, vRB-1B-infected chickens that showed no evident clinical signs were established to have a higher proportion of CD4^+^ T cells than control chickens (Fig. 5d). Moreover, vNr-c1-Meq-infected chickens showed no significant increases in the proportion of CD4^+^ T cells at any of the assessed time points (Fig. 5d), although these birds were found to have a lower proportion of Meq^+^ CD4^+^ T cells than those in the vRB-1B-infected group, with no increases observed at 14–35 dpi (Fig. 5e). Consistently, the proportions of CD4^+^ T cells among CD3^+^ T cells in the thymus and spleen followed the same group-specific trends (Fig. S4a–c). Collectively, these findings revealed no substantial generation of transformed CD4^+^ T cells in the lymphoid organs of vNr-c1-Meq-infected chickens.

### Changes in the proportions of CD8^+^ T cells and *γδ* T cells in the lymphoid organs upon infection

**Fig. 6.**
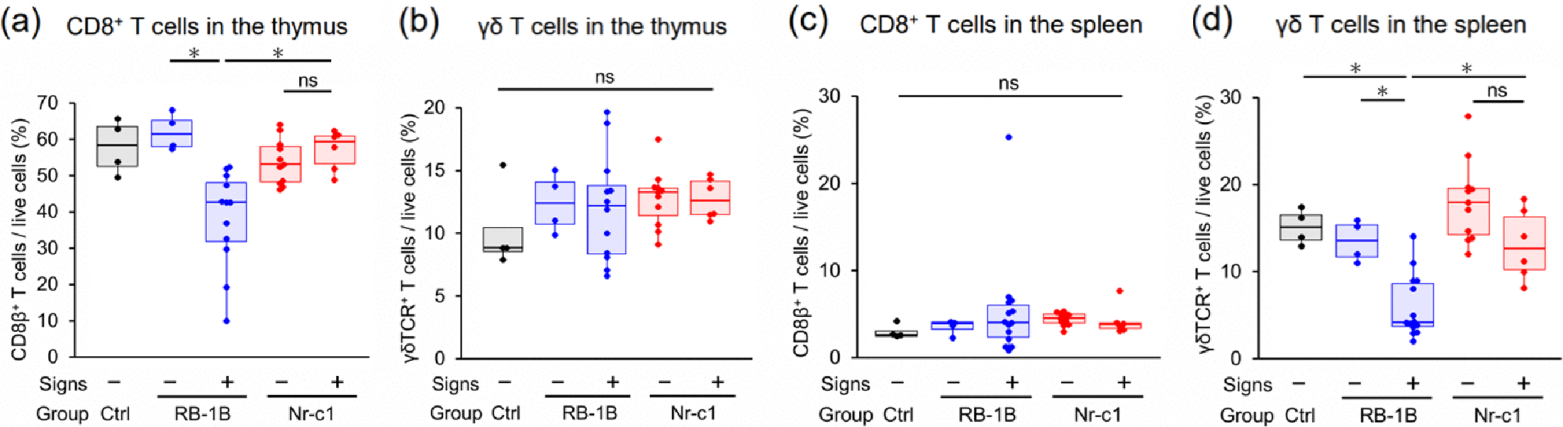
Comparison of the proportion of CD8^+^ T cells and *γδ* T cells in the thymus and spleen of chickens during disease development. (**a**)–(**d**) The proportion of (**a**) CD8^+^ T cells and (**b**) *γδ* T cells in the thymus and (**c**) CD8^+^ T cells and (**d**) *γδ* T cells in the spleen was analysed. T-cell subset proportions were compared in the spleen and thymus of rMDV-infected chickens with and without clinical signs (signs+and signs−, respectively). Samples from chickens in the control (*n*=4), vRB-1B group (signs-, *n*=4), vRB-1B group (signs+, *n*=16), vNr-c1-Meq group (signs−, *n*=11) and vNr-c1-Meq group (signs+, *n*=6) were used for these analyses. All samples were collected from chickens in animal experiment 1. Box and whisker plots showing the median (line within the box), interquartile range (box) and minimum and maximum values (whiskers). The dots represent individual data points or outliers. Asterisks indicate significant differences (*P<0.05; Steel–Dwass test).

To further investigate changes in the lymphoid organs in response to infection, for both infected groups, we analysed the proportions of CD8^+^ and *γδ* T cells among total live cells in the thymus and spleen. CD8^+^ T and *γδ* T cells play protective roles against MD [47,51], and we found that the proportions of CD8^+^ T cells in the thymus were significantly lower in vRB-1B-infected chickens with signs than in the vRB-1B-infected chickens without signs and in vNr-c1-Meq-infected chickens with signs, whereas regardless of clinical signs, we detected no significant difference in vNr-c1-Meq chickens (Fig. 6a). In contrast, there were no significant differences among the groups with respect to the proportions of *γδ* T cells in the thymus (Fig. 6b); although in the spleen, the proportions of CD8^+^ T cells showed no significant difference among groups (Fig. 6c); those of *γδ* T cells were lower in vRB-1B-infected chickens with signs (Fig. 6d). However, no similar reductions were observed in vNr-c1-Meq-infected chickens with signs (Fig. 6d). Thus, relative reductions in CD8^+^ T cells and *γδ* T cells observed in the thymus and spleen could be attributed to elevated levels of CD4^+^ T-cell transformation in vRB-1B-infected chickens. In addition, we established that among CD3^+^ T cells in the thymus and spleen, the proportions of CD8^+^ and *γδ* T cells showed similar group-specific trends (Fig. S5a–d).

### Histopathological findings in chickens infected with vNr-c1-Meq

We performed histopathological analysis to further investigate the pathology of chickens infected with vNr-c1-Meq. Additionally, we conducted IHC using monoclonal antibodies (mAbs) against Meq [45] and pp38 to detect rMDV-infected cells. Meq is highly expressed in T cells transformed by MDV, while pp38 is a lytic antigen linked to early cytolytic infection and viral reactivation [52]. In vRB-1B-infected chickens, tumour cell proliferation, characterized by mitotic activity, enlarged cell size, distinct nucleoli and Meq-positive staining, was observed in multiple organs (Table 3). Tumour lesions were also seen in the spleen, thymus, proventriculus, brain, peripheral nerves and skin in vNr-c1-Meq-infected chickens, although these were fewer and smaller than in vRB-1B-infected chickens (Table 3). Meq-positive cells were present in several organs of vNr-c1-Meq-infected chickens but were fewer than in vRB-1B-infected chickens (Table 3), suggesting reduced tumourigenicity of vNr-c1-Meq.

**Table 3.** Summary of the histopathological analysis and immunohistochemistry for Meq and pp38 in rMDV-infected chickens

Group	No.	Examination	Spleen	Bursa	Thymus	Lung	Proventri -culus	Brain	Cervical peripheral nerve	Remark
vRB-1B	1	Tumour lesions in HE	−	−	−	−	+	++	+	Gross findings: torticollis, leg paralysis Gross tumours: liver, testis
		Meq in IHC	++	+	+++	+	++	++	+	
		pp38 in IHC	−	+	−	−	+	+	−	
	2	Tumour lesions in HE	++	++	++	+++	+++	+	+	Gross tumours: spleen, kidney, ovary
		Meq in IHC	+++	++	+++	+++	+++	+	+	
		pp38 in IHC	+	+	++	+	+	+	−	
	3	Tumour lesions in HE	+	+	++	+++	+	+	−	Gross tumours: colon, cecum, lung
		Meq in IHC	++	++	++	+++	++	+	+	
		pp38 in IHC	−	−	−	+	−	−	−	
vNr-c1-Meq	1	Tumour lesions in HE	+	−	+	−	−	+	+	Gross findings: splenomegaly
		Meq in IHC	+	+	+	+	+	+	+	
		pp38 in IHC	+	+	+	+	+	+	−	
	2	Tumour lesions in HE	−	−	−	−	+	+	+	Gross findings: thymus atrophy
		Meq in IHC	+	+	+	+	+	+	+	
		pp38 in IHC	+	+	−	+	+	−	−	
	3	Tumour lesions in HE	−	−	−	−	+	−	−	Gross findings: small body weight, dirtiness of feather Lesions in HE: BALT hyperplasia
		Meq in IHC	+	−	+	+	+	−	−	
		pp38 in IHC	−	−	+	−	+	−	−	
	4	Tumour lesions in HE	−	−	+	−	+	+	+	Gross findings: thymus atrophy Lesions in HE: BALT hyperplasia
		Meq in IHC	+	+	+	+	+	+	+	
		pp38 in IHC	+	+	+	+	+	+	−	
	5	Tumour lesions in HE	−	−	−	−	−	+	−	Gross findings: splenomegaly Lesions in HE: BALT hyperplasia
		Meq in IHC	+	+	+	+	+	+	+	
		pp38 in IHC	+	−	−	−	+	+	−	

The results of tumour lesions in HE indicate the percentage of tumour lesions in the organs: +, <30%; ++, 30–80%; +++, >80%. The IHC results indicate the percentage of Meq-positive cells per five fields of view: +, <30%; ++, 30–80%; +++, >80%.

HE, hematoxylin and eosin staining.

**Fig. 7.**
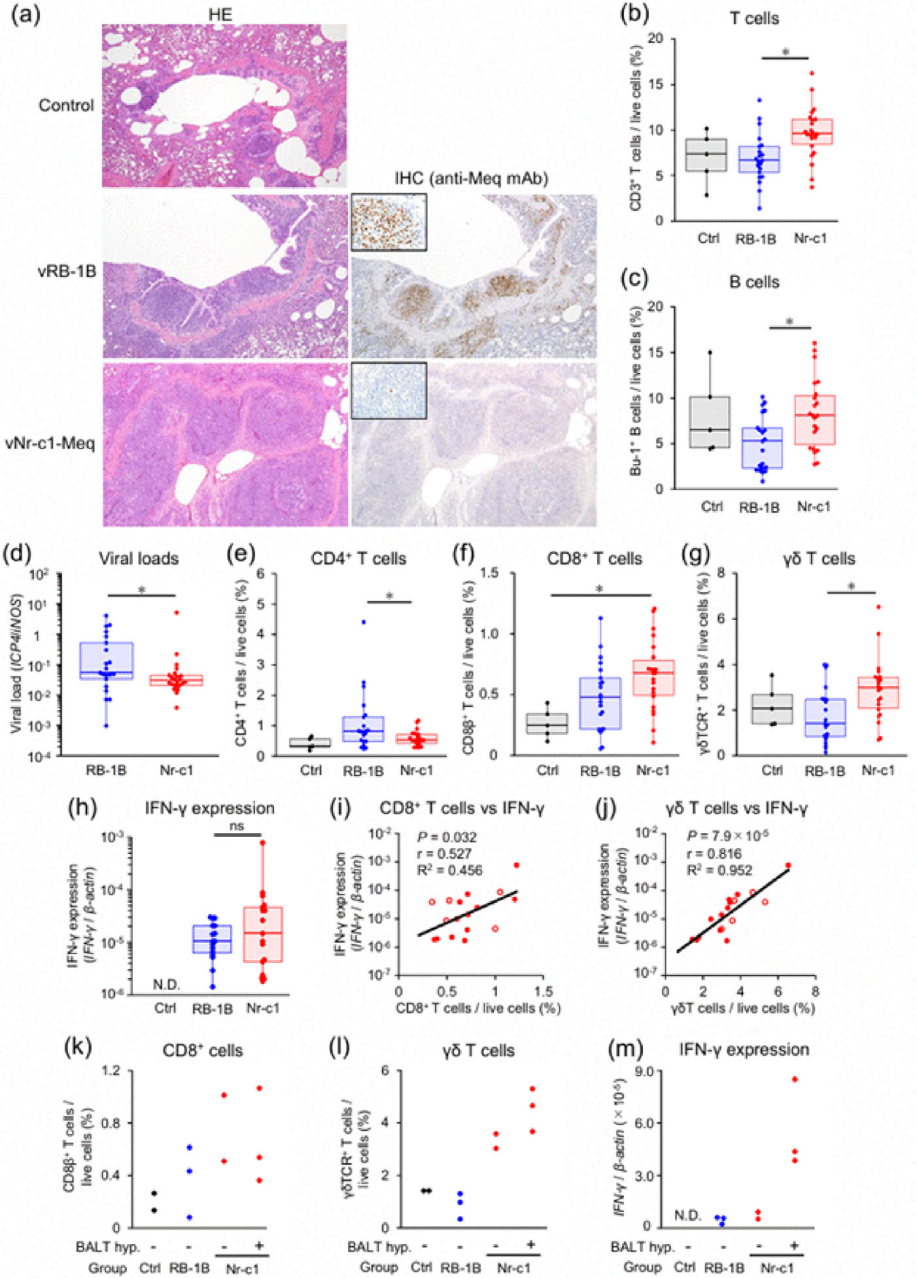
Histopathological analysis and immunological changes in the lung of rMDV-infected chickens. (**a**) Haematoxylin and eosin (HE) staining (left panels) and IHC for Meq (right panels) were performed on lung tissues from control chickens (upper panels), vRB-1B-infected chickens (middle panels) and vNr-c1-Meq-infected chickens (lower panels). All images were captured at 100× magnification. For Meq IHC, small insets in the upper left corners present images at 1,000× magnification. (**b**) Viral loads in lung tissues were compared across infected groups. (**c**)–(**g**) The proportion of (**c**) CD3^+^ T cells, (**d**) B cells, (**e**) CD4^+^ T cells, (**f**) CD8^+^ T cells and (**g**) *γδ* T cells in the live lung cells was analysed by flow cytometry. (**h**) The expression of IFN-*γ* mRNA in the lung was analysed. (**b**)–(**h**) Samples from chickens in control (*n*=5), vRB-1B-infected group (*n*=20) and vNr-c1-Meq-infected group (*n*=22) were used in these analyses. Box and whisker plots showing the median (line within the box), interquartile range (box) and minimum and maximum values (whiskers). The dots represent individual data points or outliers. (**i**)–(**j**) Relationship between (**i**) expression of IFN-*γ* mRNA and CD8^+^ T-cell proportion and (**j**) expression of IFN-*γ* mRNA and *γδ* T cell proportion was analysed in vNr-c1-Meq-infected chickens (*n*=22). Each red plot indicates values in individual chickens. Plots of chickens investigated in the histopathology are shown as red circles with white paint. *P* value, correlation coefficient (r) and coefficient of determination (r^2^) were shown in the figures. (**k**)–(**m**) Comparison of the (**k**) CD8^+^ T-cell proportion, (**l**) *γδ* T cell proportion and (**m**) expression of IFN-*γ* mRNA of chickens investigated in the histopathological analysis, control (*n*=2), vRB-1B-infected group (*n*=3) and vNr-c1-Meq-infected group (*n*=5). The Nr-c1 group was divided into chickens without BALT hyperplasia (BALT hyp. −) (*n*=2) and with BALT hyperplasia (BALT hyp. +) (*n*=3). (**a**)–(**m**) All data were obtained from chickens in animal experiment 2. Asterisks indicate significant differences [*P<0.05; (**d**) Mann–Whitney U test, (**b**), (**c**), (**e**)–(**h**) Steel–Dwass test].

Notably, three of five samples from vNr-c1-Meq-infected chickens subjected to histopathological analysis exhibited hyperplasia of BALT, characterized by lymphocyte infiltration and a few Meq-positive cells in the lungs (Table 3, Fig. 7a). In lung samples from all chickens, PCR and real-time PCR were negative for *M. gallisepticum*, *M. synoviae*, *P. multocida*, *B. avium*, *E. coli*, infectious laryngotracheitis virus, infectious bronchitis virus, Newcastle disease virus and avian influenza virus (data not shown), which can cause BALT hyperplasia [53,55]. In contrast, vRB-1B-infected chickens developed tumour lesions with Meq-positive cell accumulation in the lungs, while no BALT hyperplasia was observed (Table 3, Fig. 7a). pp38-positive cells were also present in the lungs of both groups, but their numbers did not differ significantly (Fig. S6a). Thus, a few infected (or transformed) cells were present in the lungs of vNr-c1-Meq-infected chickens, and lymphocyte infiltration with BALT hyperplasia was considered a sign of severe inflammation. Collectively, these data suggest that lung inflammation may contribute to open-mouth breathing in chickens infected with vNr-c1-Meq.

### Characteristics of T-cell subsets in the lungs of chickens infected with vNr-c1-Meq

To investigate the lymphocyte populations associated with BALT hyperplasia in vNr-c1-Meq-infected chickens, we first analysed the proportions of T and B cells among viable lung cells. The proportion of T cells was higher in vNr-c1-Meq-infected chickens (Fig. 7b), whereas B-cell proportions were lower in vRB-1B-infected chickens (Fig. 7c). We also evaluated T-cell subsets in the lungs of both infected groups. The proportions of CD4^+^, CD8^+^ and *γδ* T cells within CD3^+^ T cells did not exhibit distinguishing features in vNr-c1-Meq-infected chickens (Fig. S6b–d). However, both viral load and CD4^+^ T-cell proportions in viable lung cells were lower in vNr-c1-Meq-infected chickens than in vRB-1B-infected chickens (Fig. 7d and e). These findings suggest group-level differences in the number of infected (or transformed) T cells, consistent with histopathological results (Fig. 7a). Conversely, the proportions of CD8^+^ T cells and *γδ* T cells in viable lung cells were higher in vNr-c1-Meq-infected chickens (Fig. 7f and g), suggesting a more robust immune response against vNr-c1-Meq infection.

Next, we examined mRNA expression of several proinflammatory cytokines, including *IL-1β*, *IL-6*, *TNF-α* and *IFN-γ* in the lungs, but no marked trends were observed in expression levels between the infected groups (data not shown). However, high IFN-*γ* mRNA expression was noted in the lungs of some vNr-c1-Meq-infected chickens (Fig. 7h). Additionally, IFN-*γ* mRNA expression showed a moderate positive correlation with CD8^+^ T-cell proportion (correlation coefficient=0.527) (Fig. 7i) and a strong positive correlation with *γδ* T-cell proportion (correlation coefficient=0.816) (Fig. 7j). Similarly, elevated IFN-*γ* concentrations were found in the plasma of some vNr-c1-Meq-infected chickens (Fig. S6e). Plasma IFN-*γ* concentration showed no correlation with CD8^+^ T-cell proportion (Fig. S6f) but demonstrated a positive correlation with *γδ* T cell proportion (correlation coefficient=0.669) (Fig. S6g). Among chickens evaluated histopathologically, increased proportions of CD8^+^ and *γδ* T cells were detected in vNr-c1-Meq-infected individuals (Fig. 7k and l). Notably, the *γδ* T cell proportion was higher in vNr-c1-Meq-infected chickens with BALT hyperplasia (Fig. 7l). Furthermore, these chickens exhibited elevated IFN-*γ* mRNA expression (Fig. 7m) and increased IFN-*γ* concentration (Fig. S6h). Taken together, the increased *γδ* T cell proportion in the lungs of vNr-c1-Meq-infected chickens may be associated with BALT hyperplasia and may contribute to the observed inflammatory response.

## Discussion

Marek’s disease (MD) is currently effectively controlled by vaccination; however, sporadic cases of MD continue to be reported, and circulating MDV field strains show a tendency towards increased virulence. Distinct polymorphisms are known to exist in the *meq* oncogenes of various MDV strains and are associated with differences in virulence [22]. In Japan, sporadic MD outbreaks have occurred in vaccinated chickens, and MDV strains circulating locally exhibit characteristic amino acid polymorphisms [27]. In this study, we examined how Meq polymorphisms in Japanese isolates influence transcriptional regulation and MDV pathogenicity. Meq from Japanese isolates exhibited weaker transcriptional regulation than RB-1B-Meq. In addition, vNr-c1-Meq-infected chickens demonstrated slower disease progression than vRB-1B-infected chickens and lacked gross tumour lesions, consistent with lower viral loads and reduced CD4^+^ T-cell proportions in lymphoid tissues. Intriguingly, however, vNr-c1-Meq-infected chickens exhibited atypical clinical signs such as open-mouth breathing, likely attributable to BALT hyperplasia in the lungs. Furthermore, these chickens with BALT hyperplasia showed higher *γδ* T cell proportions and elevated IFN-*γ* mRNA expression in the lungs. Collectively, these findings suggest that Meq polymorphisms in Japanese MDV isolates influence viral pathogenesis.

Meq from Japanese isolates exhibited reduced transrepression on the *pp38* promoter compared to RB-1B-Meq. Transrepression of viral lytic genes such as pp38 may be linked to the establishment of MDV latency. Although no differences in viral loads were observed between the two rMDV-infected groups, higher titres of vNr-c1-Meq were recovered from infected chickens at 14 dpi in the reactivation assay, suggesting a delay in the establishment of latency in vNr-c1-Meq-infected chickens. Thus, reduced efficiency in establishing latency may partially underlie the diminished tumourigenic potential of vNr-c1-Meq. Additionally, Meq from Japanese isolates exhibited lower transactivation activity than RB-1B-Meq, potentially contributing to the decreased tumourigenicity of vNr-c1-Meq. As previously reported [27], amino acid residues at positions 77 and 80 in the bZIP domain influence Meq transactivation capacity. In the present study, the lysine-aspartate combination at positions 77 and 80 in RB-1B-Meq showed higher transactivation activity, whereas the glutamate-tyrosine combination found in Japanese isolates corresponded to lower activity. Therefore, polymorphisms in the BR may contribute to reduced transactivation activity, leading to mild disease progression in vNr-c1-Meq-infected chickens. Conversely, substitutions at positions 176 and 217 in RB-1B-Meq, which disrupt the PPPP motifs, enhanced transactivation activity, consistent with previous findings [56]. Thus, domain-specific Meq polymorphisms may modulate MD virulence by altering transcriptional regulation, dimerization partner preference or nuclear subdomain localization [16]. However, to further clarify the virulence impact of Japanese Meq, the pathogenicity of Japanese MDV strains must be directly assessed and compared to that of vNr-c1-Meq.

Histopathological examination confirmed tumour lesions in vNr-c1-Meq-infected chickens, although only a few Meq-positive cells were observed, and gross tumours were absent. Tumour severity in the vNr-c1-Meq-infected group was lower than in the vRB-1B-infected group, consistent with lower viral loads and CD4^+^ T-cell proportions in the thymus and spleen. These findings suggest a less efficient or delayed tumour development in vNr-c1-Meq-infected chickens compared to the vRB-1B-infected chickens, which is consistent with its reduced tumourigenicity. *In vitro* characterization of the transformation capacity of Meq of Japanese isolates further supports its reduced tumourigenicity compared to RB-1B-Meq. Nr-c1, the Japanese isolate encoding the Meq used in this study, previously caused MD with neurological signs and solid tumours in vaccinated farm chickens. In contrast, another Japanese isolate, Kgs-c1, which encodes a Meq protein identical to that of Nr-c1, caused visceral tumours in poultry farm chickens and showed slower disease progression than vv+MDV in the experimental infection [28]. Thus, clinical signs such as neurologic dysfunction and visceral tumours may manifest later in vNr-c1-Meq-infected chickens under extended observation. Whole-genome sequence of Kgs-c1 also presents distinct features in several viral genes, including *ICP4* and *UL36*, in addition to the *meq* gene [22]. Therefore, tumour development in chickens infected with Nr-c1 or Kgs-c1 may also be influenced by other viral determinants. To confirm the roles of these additional factors in pathogenesis, animal challenge experiments using rMDVs derived from Japanese isolates encoding RB-1B-Meq are warranted.

Most importantly, replacement of RB-1B-Meq in vRB-1B with Nr-c1-Meq resulted in a distinct clinical sign, open-mouth behaviour, in infected chickens. Two potential causes were considered: respiratory or gastrointestinal abnormalities. BALT hyperplasia has been reported in avian respiratory infections such as mycoplasmosis and infectious bronchitis [53,55]. Conversely, literature suggests that vagus nerve dysfunction due to lymphocyte infiltration may lead to crop paralysis and gasping in chickens infected with MDV [57]. In this study, histopathology revealed BALT hyperplasia in some vNr-c1-Meq-infected chickens; however, these birds did not show respiratory signs at the time of sampling. In contrast, no differences were observed in MD lesions of cervical peripheral nerves between the rMDV-infected groups. Additionally, tumour lesions with Meq-positive cells were more prominent in the proventriculus of vRB-1B-infected chickens compared to vNr-c1-Meq-infected chickens. Thus, severe BALT hyperplasia may cause pulmonary stenosis and be involved in respiratory signs, although these data do not establish a solid relationship between the BALT hyperplasia and open-mouth breathing. In contrast, vNr-c1-Meq-infected chickens exhibited thymus atrophy, potentially reducing immune responses. Immunocompromised chickens can acquire opportunistic infections and may exhibit unexpected clinical manifestations. Consequently, the possibility that unidentified environmental factors were involved in BALT hyperplasia or the respiratory signs cannot be completely ruled out, despite the PCR screening results for major avian respiratory pathogens being negative. However, we have previously confirmed that the introduction of the amino acid combination glutamate and tyrosine at positions 77 and 80 in the Meq sequence of RB-1B-based rMDV resulted in a reduction in virulence and changes in clinical manifestations, including open-mouth breathing associated with BALT hyperplasia [26]. Furthermore, cases of MD with open-mouth breathing have recently been reported in vaccinated layer flocks in Japan [58], and, except threonine and phenylalanine at positions 254 and 258, respectively, the amino acid sequences of Meq in these birds resembled those in Nr-c1-Meq [59]. Although BALT hyperplasia was not confirmed in these chickens, MD cases with similar clinical features appear to be emerging under field conditions.

IFN-*γ* inhibits MDV replication *in vitro* and suppresses MD progression in infected chickens [60]. Additionally, MD vaccination induces IFN-*γ*^+^ *γδ* T cells in the lung [61]. In contrast, IFN-*γ* expression in *γδ* T cells is suppressed within the tumour microenvironment of MDV-infected chickens [62]. Thus, IFN-*γ*^+^ *γδ* T cells play critical roles in limiting MD progression. In this study, we observed increased *γδ* T-cell proportions in the lungs of vNr-c1-Meq-infected chickens and a strong positive correlation between *γδ* T-cell proportion and IFN-*γ* mRNA expression. Additionally, *γδ* T-cell proportion and IFN-*γ* mRNA expression were associated with BALT hyperplasia. Given the effector function of *γδ* T cells in controlling MD development [47,63], increased *γδ* T-cell numbers may suppress tumour cell proliferation in vNr-c1-Meq-infected chickens. Similarly, CD8^+^ T-cell proportions, another population with a protective role against MD development [51], were elevated in the lungs of vNr-c1-Meq-infected chickens. However, the CD8^+^ T-cell increase occurred independently of BALT hyperplasia and did not correlate with IFN-γ concentration. Thus, the increase in effector cells in lungs with BALT hyperplasia, particularly *γδ* T cells, appeared to be associated with IFN-γ expression; this is reminiscent of severe pulmonary inflammation. Infectious virus was abundantly present in vNr-c1-Meq-infected chickens, even at 14 dpi, suggesting that immune responses against infected cells may be more strongly induced or sustained longer. Additionally, stimulation via Toll-like receptor 3 induces IFN-*γ* production in pulmonary *γδ* T cells in infected chickens, although further signalling from other immune cell types is also required [64]. Therefore, differences in viral antigen burden and immune response magnitude may have influenced disease outcomes. Further investigation is needed to clarify the mechanisms driving *γδ* T cell expansion and to understand why lesions remained confined to the lungs of vNr-c1-Meq-infected chickens.

In this study, we demonstrated that a few amino acid substitutions in Meq can alter the pathogenesis of MD, consistent with previous findings [22,65]. Nevertheless, a limitation of the study is that the survival rate varied between the two animal experiments, presumably due to multiple unidentified factors, including the shorter experimental period and environmental factors, although we observed similar differences in clinical signs. We assessed the correlation between IFN-*γ* expression and *γδ* T cells or CD8^+^ T cells in the lungs of vNr-c1-Meq-infected chickens; however, the association between IFN-γ expression and BALT hyperplasia or respiratory signs requires cautious interpretation due to the small number of birds with data available for virological and histopathological findings. In addition, other cell types, including macrophages and natural killer (NK) cells, also produce IFN-*γ* and may contribute to MD resistance [66,67]. We could not analyse NK cell populations because a specific marker for chicken NK cells is unavailable. NK cells may be enriched within the CD45^+^ CD3⁻ Bu-1⁻ cell population. Analyses using IFN-*γ* and the degranulation marker CD107a may facilitate the identification of macrophage and NK cell populations exhibiting an activated phenotype. Additionally, we could not compare the properties of transformed cells between vRB-1B- and vNr-c1-Meq-infected chickens due to the absence of gross tumours in vNr-c1-Meq-infected chickens. Single-cell transcriptomic profiling of isolated T cell populations may clarify the mechanisms underlying the divergent pathogenesis in future studies.

In conclusion, this study investigated the effects of polymorphisms in Meq of Japanese isolates on pathogenicity and revealed that these polymorphisms decreased the pathogenicity of MDV. Intriguingly, rMDV encoding Meq from Japanese isolates induced open-mouth breathing, likely due to pulmonary alterations associated with BALT hyperplasia. Additionally, BALT hyperplasia tended to correlate with the proportion of *γδ* T cells and IFN-*γ* expression. These findings suggest that Meq polymorphisms in Japanese isolates may mitigate the virulence of MDV and contribute to atypical clinical manifestations. *meq* genes in most Japanese strains are genetically related to those found in Europe, Asia and Africa [4,5, 29,35, 68]. MD cases presenting as open-mouth breathing have been reported in Japan; therefore, cases with similar clinical features may be occurring in other countries and may be under-recognized. To assess and prevent potential economic losses due to MD, continued investigation of field cases, clinical signs and viral genomic characteristics is warranted across different regions.

## Supplementary material

10.1099/jgv.0.002232 Uncited Supplementary Material 1.

10.1099/jgv.0.002232 Uncited Supplementary Material 2.

## Abbreviations

BAC: bacterial artificial chromosome
BALT: bronchus-associated lymphoid tissues
BR: basic region
bZIP: basic leucine zipper
CEFs: chicken embryo fibroblasts
DMEM: Dulbecco’s Modified Eagle’s Medium
dpi: days post-infection
ICP4: infected cell protein 4
IHC: immunohistochemistry
IRL: internal repeat long
MD: Marek’s disease
MDV: Marek’s disease virus
NK: natural killer
PBMCs: peripheral blood mononuclear cells
PBS-T: PBS containing 0.5% Tween 20
PRRs: proline-rich regions
qPCR: quantitative PCR
RFLP: restriction fragment length polymorphism
rMDV: recombinant Marek’s disease virus
TD: transactivation domain
TRL: terminal repeat long
ZIP: leucine zipper domain
