# Supplementary material for: Virulence of Marek’s disease virus in Japan is linked to polymorphisms in the meq oncogene
Source: J Gen Virol. 2026 Feb 19;107(2):002232. doi: 10.1099/jgv.0.002232 (PMC12919941; doi:10.1099/jgv.0.002232)
Supplement: Uncited Supplementary Material 1. [file jgv-107-02232-s001.pdf]

S1 Fig

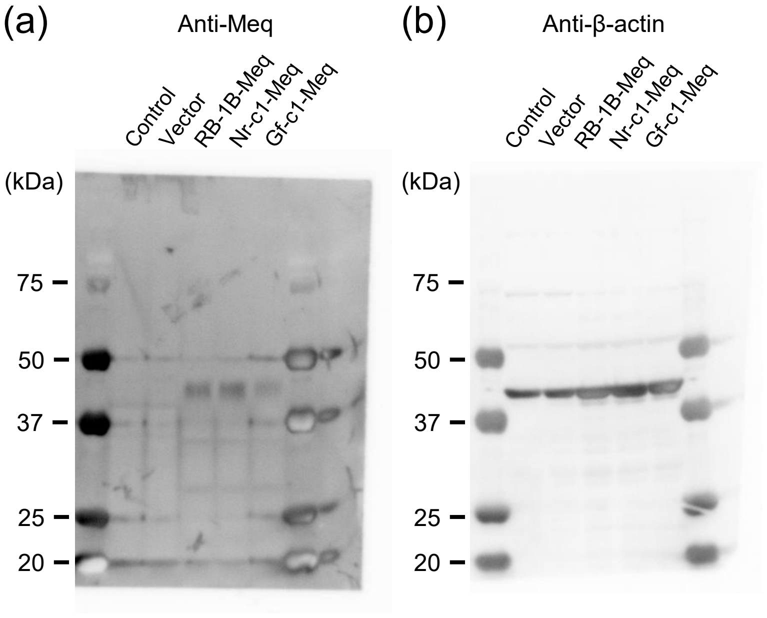

S1 Fig. Expression of Meq in stable cell lines.

Meq expression was analyzed in DF-1 stably expressing RB-1B-Meq, Nr-c1-Meq, or Gf-c1-Meq by western blotting. Non-transfected DF-1 and pCI-neo vector transfected cells were used as negative controls. Heat-denatured samples were loaded to SDS-PAGE followed by immunoblotting with (a) anti-Meq antisera or (b) anti-β-actin monoclonal antibody as a loading control. Representative results from two independent experiments are shown.

S2 Fig

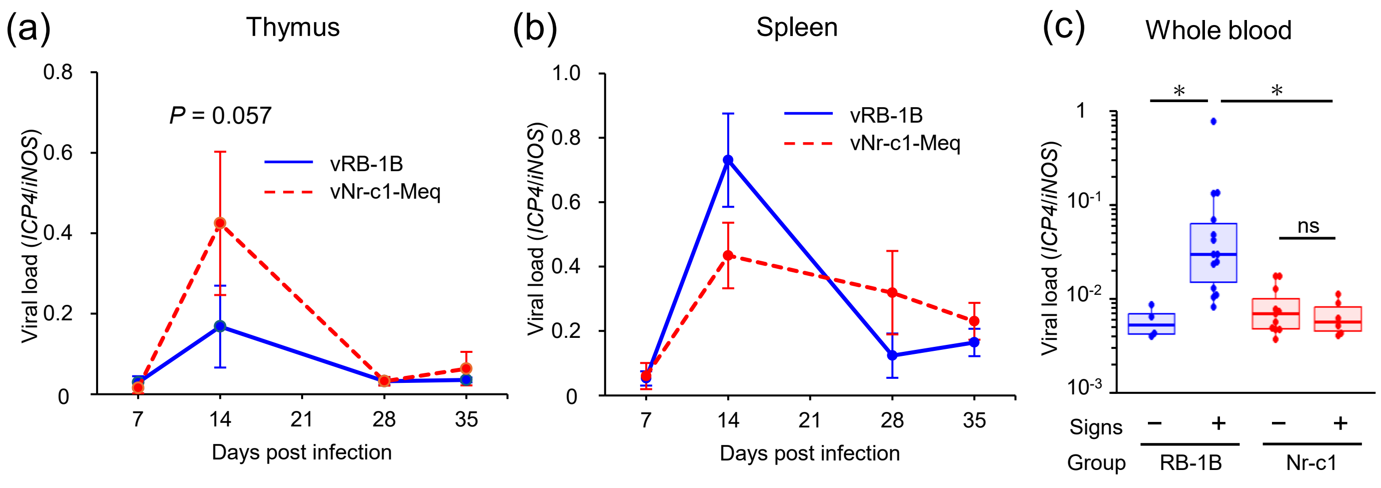

S2 Fig. Growth kinetics of recombinant MDVs (rMDVs) *in vivo*.

Growth kinetics of rMDVs in (a) thymus and (b) spleen were analyzed by qPCR. Error bars indicate standard deviations. (e) Viral loads in whole blood from rMDV-infected chickens in animal experiment 1 were analyzed by qPCR. Viral loads were compared between rMDV-infected chickens with and without signs. Statistical analysis was performed by. Asterisks indicate significant differences (*p < 0.05; (a)–(b) Mann-Whitney U test, (c) Steel-Dwass test).

S3 Fig

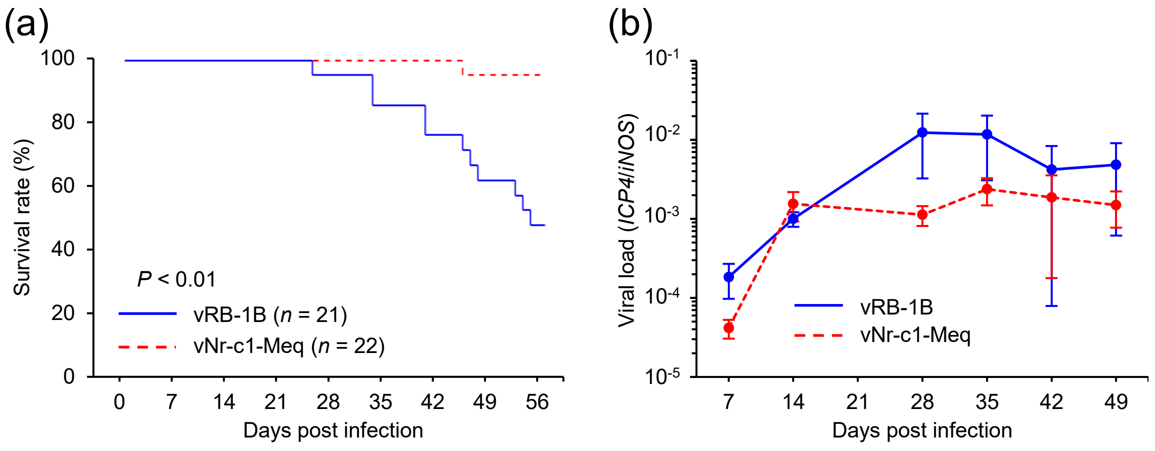

S3 Fig. Pathogenicity of vNr-c1-Meq in the animal experiment 2

(a) Chickens that showed clinical signs were euthanized as the humane endpoint, and Kaplan-Meier survival curves were generated. The log-rank test was conducted to compare the survival rate of chickens infected with each rMDV. $p < 0.05$ was considered as statistically significant. (b) Growth kinetics of rMDVs in whole blood were analyzed by qPCR. Error bars indicate standard deviations. Statistical analysis was performed by the Mann-Whitney U test.

S4 Fig

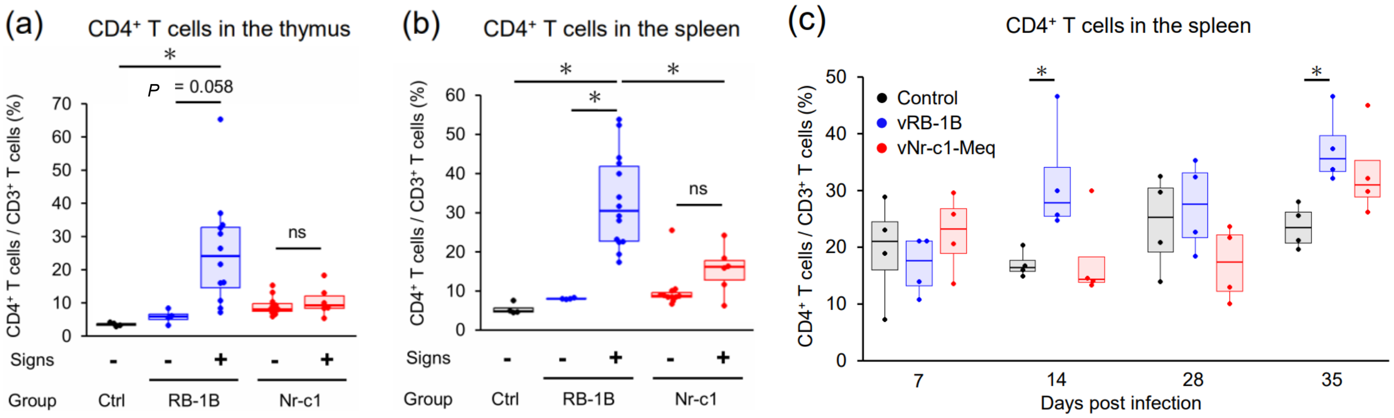

S4 Fig. Comparison of proportion of CD4⁺ T cells and Meq⁺ CD4⁺ T cells in the thymus and spleen of chickens at disease development

The proportion of (a) $CD4^+$ T cells in the thymus, (b) $CD4^+$ T cells in the spleen were analyzed. In (a, b), the proportions of T cell subsets were compared in the spleen and thymus of rMDV-infected chickens with and without signs. (c) $CD4^+$ T cells in the spleen were analyzed at 7, 14, 28 and 35 dpi. (a)–(c) Box-and-whisker plots showing the median (line within the box), interquartile range (box), and minimum and maximum values (whiskers). The dots represent individual data points or outliers. Asterisks indicate significant differences (*$p < 0.05$; (a), (b) Steel-Dwass test, (c) Mann-Whitney U test).

S5 Fig

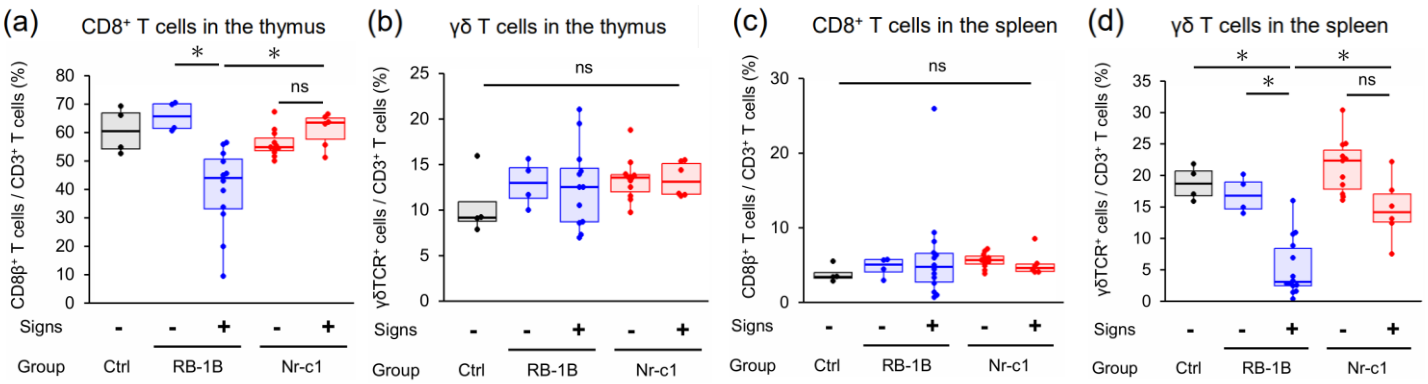

S5 Fig. Comparison of proportion of CD8⁺ and γδ T cells in the thymus and spleen of chickens at disease development

The proportion of (a) $CD8^+$ T cells in the thymus, (b) γδ T cells in the thymus, (c) $CD8^+$ T cells in the spleen, and (d) γδ T cells in the spleen were analyzed. In (a–d), the proportions of T cell subsets were compared in the spleen and thymus of rMDV-infected chickens with and without signs. (a)–(d) Box-and-whisker plots showing the median (line within the box), interquartile range (box), and minimum and maximum values (whiskers). The dots represent individual data points or outliers. Asterisks indicate significant differences (*$p < 0.05$; Steel-Dwass test).

S6 Fig

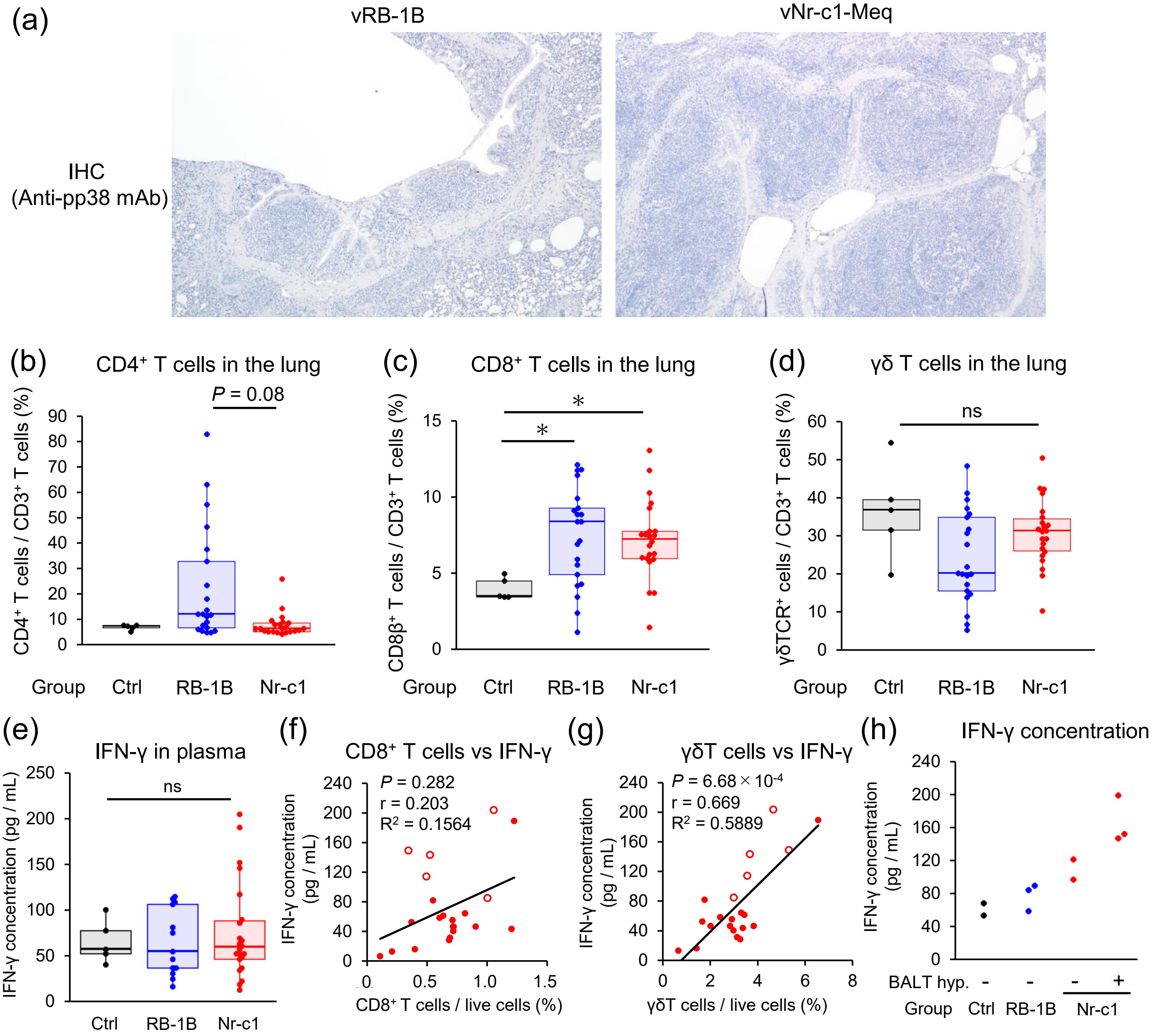

(a)
vRB-1B
vNr-c1-Meq
IHC
(Anti-pp38 mAb)
(b)
CD4⁺ T cells in the lung
P = 0.08
CD4⁺ T cells / CD3⁺ T cells (%)
Group Ctrl RB-1B Nr-c1
(c)
CD8⁺ T cells in the lung
*
*
CD8β⁺ T cells / CD3⁺ T cells (%)
Group Ctrl RB-1B Nr-c1
(d)
γδ T cells in the lung
ns
γδTCR⁺ cells / CD3⁺ T cells (%)
Group Ctrl RB-1B Nr-c1
(e)
IFN-γ in plasma
ns
IFN-γ concentration (pg / mL)
Ctrl RB-1B Nr-c1
(f)
CD8⁺ T cells vs IFN-γ
P = 0.282
r = 0.203
R² = 0.1564
IFN-γ concentration (pg / mL)
CD8⁺ T cells / live cells (%)
(g)
γδT cells vs IFN-γ
P = 6.68 × 10⁻⁴
r = 0.669
R² = 0.5889
IFN-γ concentration (pg / mL)
γδT cells / live cells (%)
(h)
IFN-γ concentration
IFN-γ concentration (pg / mL)
BALT hyp. - - - +
Group Ctrl RB-1B Nr-c1

S6 Fig. Immunohistochemistry for pp38 in the lung of rMDV-infected chickens.

(a) Immunohistochemistry (IHC) for pp38 in the lung from chickens infected with vRB-1B (left panel) and vNr-c1-Meq (right panel). (b) $CD4^+$ T cells, (c) $CD8^+$ T cells and (d) γδ T cells in the $CD3^+$ T cells in the lung were analyzed. In (b–d), the proportions of T cell subsets were compared in the lungs of rMDV-infected chickens with and without disease. (e) IFN-γ concentration in plasma were analyzed. (b)–(e) Box-and-whisker plots showing the median (line within the box), interquartile range (box), and minimum and maximum values (whiskers). The dots represent individual data points or outliers. (f)-(g) Relationship between (f) IFN-γ concentration in plasma and $CD8^+$ T cell proportion and (g) IFN-γ concentration in plasma and γδ T cell proportion were analyzed in vNr-c1-Meq-infected chickens. Each red plot indicates values for individual chickens. Plots of chickens examined by histopathology are shown as red circles with white paint. (h) IFN-γ concentration in plasma of chickens examined in the histopathological analysis. Nr-c1 group was divided into chickens without BALT hyperplasia (BALT hyp. -) and with BALT hyperplasia (BALT hyp. +). p value, correlation coefficient (r), and coefficient of determination (R^2) were shown in figures. Asterisks indicate significant differences (*$p < 0.05$; Steel-Dwass test).

S7 Fig

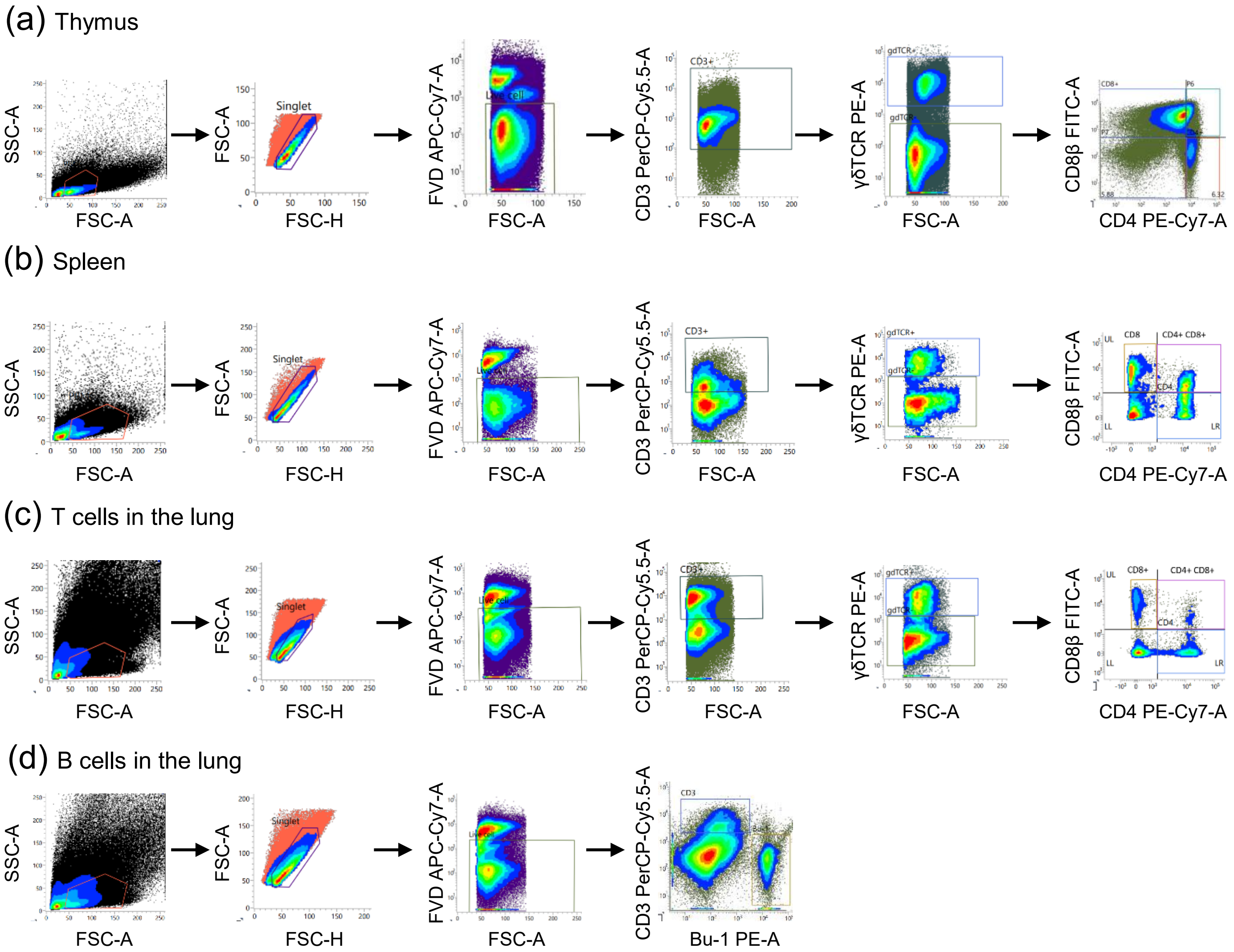

S7 Fig. Gating strategy for the analysis of the proportion of T cell subsets.

A representative gating strategy is shown for analyzing the proportions of γδ T cells, CD8β^{+} T cells, and CD4^{+} T cells in (a) the thymus, (b) spleen, and (c) lung and (d) proportion of B cells (Bu1^{+}) in the lung. Dead cells were excluded using Fixable Viability Dye APC/Cy7.
